# Stress granule clearance mediated by V-ATPase-interacting protein NCOA7 mitigates ovarian aging

**DOI:** 10.1038/s43587-025-00927-w

**Published:** 2025-07-31

**Authors:** Ting Dong, Nianyu Li, Huirui Wang, Hanbing Zhu, Yinghui Gao, Yue Liu, Fang Fang, Xiaojie Fu, Pinxin Si, Cheng Li, Mei Li, Fei Wang, Shidou Zhao, Ting Guo, Linlin Cui, Xinyi Jiang, Xiaohui Liu, Han Zhao, Yingying Qin, Zi-Jiang Chen, Hongxiang Lou, Xue Jiao

**Affiliations:** 1 Department of Natural Products Chemistry, Key Lab of Chemical Biology of the Ministry of Education, Shandong University, Jinan, China; 2 Institute of Medicinal Biotechnology, Chinese Academy of Medical Sciences and Peking Union Medical College, Beijing, China; 3 State Key Laboratory of Reproductive Medicine and Offspring Health, Center for Reproductive Medicine, Institute of Women, Children and Reproductive Health, the Second Hospital, Shandong University, Jinan, China; 4 National Research Center for Assisted Reproductive Technology and Reproductive Genetics, Shandong University, Jinan, China; 5 Key Laboratory of Reproductive Endocrinology (Shandong University), Ministry of Education, Jinan, China; 6 Shandong Technology Innovation Center for Reproductive Health, Jinan, China; 7 Shandong Provincial Clinical Research Center for Reproductive Health, Jinan, China; 8 Shandong Key Laboratory of Reproductive Research and Birth Defect Prevention, Jinan, China; 9 Research Unit of Gametogenesis and Health of ART-Offspring, Chinese Academy of Medical Sciences (No.2021RU001), Jinan, China; 10 The Affiliated Suzhou Hospital of Nanjing Medical University, Suzhou Municipal Hospital, Gusu School, Nanjing Medical University, Nanjing, China; 11 Department of Reproductive Medicine, Department of Obstetrics and Gynecology, Shandong Provincial Hospital Affiliated to Shandong First Medical University, Jinan, China; 12 NMPA Key Laboratory for Technology Research and Evaluation of Drug Products and Key Laboratory of Chemical Biology (Ministry of Education), Department of Pharmaceutics, School of Pharmaceutical Sciences, Cheeloo College of Medicine, Shandong University, Shandong, China; 13 State Key Laboratory of Bioactive Substance and Function of Natural Medicines, Institute of Materia Medica, Chinese Academy of Medical Sciences and Peking Union Medical College, Beijing, China; 14 Shanghai Key Laboratory for Assisted Reproduction and Reproductive Genetics, Shanghai, China; 15 Department of Reproductive Medicine, Ren Ji Hospital, Shanghai Jiao Tong University School of Medicine, Shanghai, China

**Keywords:** Reproductive disorders, Ageing

## Abstract

Reproductive longevity is essential for female fertility and healthy aging; however, the role of stress response, especially stress granule accumulation, in ovarian aging remains elusive and interventions are lacking. Here, we identified deleterious mutations and decreased expression of NCOA7, a stress-response protein related to granulosa cell senescence in women with physiological and pathological ovarian aging. NCOA7 deletion accelerates oxidative stress-related cellular senescence, ovarian aging and fecundity decline in mice. Mechanistically, NCOA7 partitions into the stress granule containing G3BP1–V-ATPase and facilitates autophagic degradation of stress granules to relieve stress. Boosting granulophagy with rapamycin or lipid nanoparticle-based mRNA delivery of *NCOA7* accelerates stress granule clearance, alleviating cellular senescence in human granulosa cells and delaying ovarian aging in mice. This study depicts a mechanism for ovarian resilience to stress and provides potential targets for therapeutic strategies to alleviate ovarian aging.

## Abstract (web summary)

The role of cellular stress responses in ovarian aging remains incompletely understood. In this study, the authors identify NCOA7 as a facilitator of autophagic clearance of stress granules in granulosa cells, conveying protection against ovarian aging. Loss of NCOA7 promotes ovarian aging, whereas its expression via mRNA delivery delays aging.

## Main

Aging is almost universal among organisms and underlies chronic disease as well as tissue deterioration and cellular senescence^1,2^. The ovary is one of the first organs to age in humans, with functional decline occurring decades before other somatic organ systems^3,4^. In addition to age-associated physiological ovarian aging, genetic, autoimmune, iatrogenic or environmental stimuli could also accelerate ovarian aging and thus trigger premature ovarian insufficiency (POI)^5–7^. Ovarian aging is characterized by a reduction in oocyte quantity and quality, which together contribute to increased incidences of infertility, miscarriage, pregnancy complications and birth defects^4,8^. Ovarian aging also causes hormonal deficiency that systemically impacts other organs, ultimately adversely affecting overall health, longevity and socioeconomic equality^4^. These repercussions are becoming globally prevalent as increasing childbearing age and post-menopausal lifespan increase, suggesting an urgent need for interventions that improve quality of life by alleviating ovarian aging.

The ovary is vulnerable to oxidative stress, which has long been considered a major contributor to ovarian aging^9–11^. Yet the mechanism by which oxidative stress drives ovarian aging remains unclear. Stress granules (SGs), which are transient membraneless structures formed upon oxidative stress, have emerged as a key protective mechanism to relieve stress damage and promote cell survival^12,13^. Disruption of SG-mediated stress responses has been shown to increase vulnerability to stress in multiple aging-related diseases^14–16^. Conversely, some chronic stresses are known to result in aberrant SG accumulation, accelerated aging and/or aging-associated diseases, including neurodegenerative disorders and cancer, suggesting SG accumulation as a potential target for aging interventions^17–20^. Considerable advances have been made in our understanding of SG assembly, while the mechanisms governing their elimination remain controversial, especially the role of autophagy-dependent clearance. A plethora of evidence indicates that short-lived SGs undergo ubiquitin-mediated disassembly^21–23^. SG autophagy is still poorly understood, but some insight has been gained, such as the typical autophagic cargoes Cdc48 (also known as VCP) and HDAC6, as well as the role of p62 in SGs^19,24–26^. Whether disturbance in SG metabolism, if any, is implicated in ovarian aging, and whether targeting autophagic degradation of persistent SGs serves as potential intervention for ovarian aging, remain unknown.

Here, we identified deleterious mutations and reduced expression of *NCOA7*, a typical member of TBC and lysine motif (LysM) domain containing (TLDc) family to resist oxidative stress, which correlated with cellular senescence of human ovarian granulosa cells (GCs), during both physiological and pathological ovarian aging. NCOA7 deficiency in mice promotes oxidative stress-related cellular senescence of ovarian GCs and accelerates fertility decline and ovarian aging. Mechanistically, NCOA7 is required for G3BP1–V-ATPase complex interaction that mediates autophagic clearance of accumulated SGs in the cytoplasm to relieve stress. Moreover, rapamycin treatment to boost granulophagy or nanotherapeutic upregulation of NCOA7 accelerates SG removal, alleviating senescence in human GCs and delaying ovarian aging in mice. Our findings reveal an unrecognized mechanism underlying female reproductive longevity and provide potential therapeutic targets for ovarian aging.

## Results

### POI-associated *NCOA7* mutations enhance cellular senescence

**Fig. 1:**
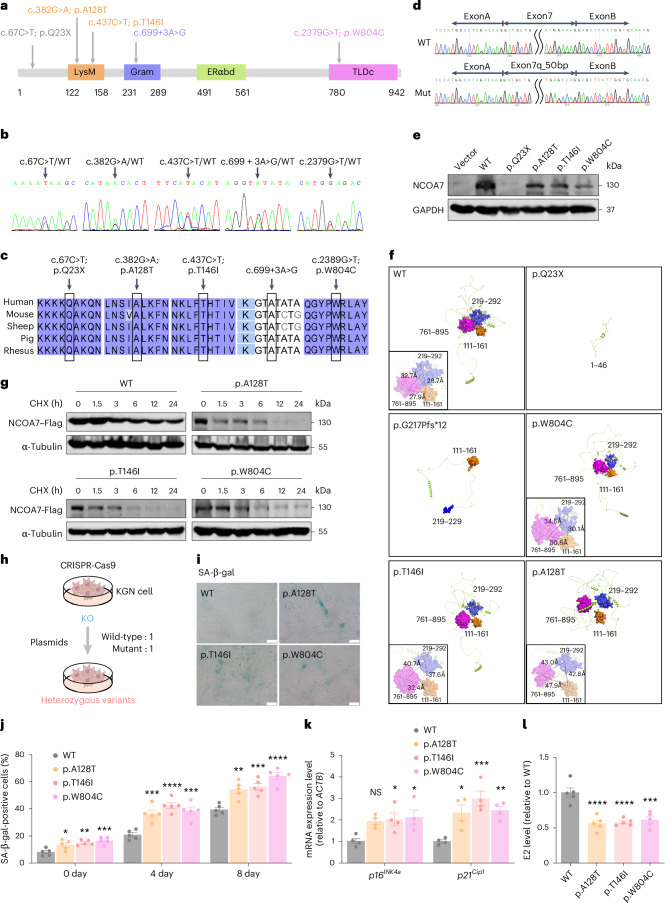
*NCOA7* variants identified in individuals with POI are associated with increased cellular senescence. **a**, A schematic diagram showing the domain architecture of the NCOA7 protein and the position of variations identified in individuals with POI. **b**, Sanger sequencing showing the five *NCOA7* heterozygous variants. **c**, Alignment of NCOA7 protein sequences across multiple mammals, highlighting the conserved amino acids at NCOA7 variants in the frame. **d**, Sequence analysis of the c.699+3A>G variant by minigene assay. Mut, mutation. **e**, Western blot of NCOA7 protein levels in wild-type (WT) and *NCOA7* mutant (Mut) KGN cells. **f**, Predicted three-dimensional structures of the WT and mutant NCOA7 proteins. An enlarged view of the structural changes of the trimer-like region is shown in the lower left corner, with intervals between each subunit: WT (32.7 Å, 28.7 Å, 27.9 Å), p.A128T (43.0 Å, 47.9 Å, 42.8 Å), p.T146I (32.4 Å, 37.6 Å, 40.7 Å) and p.W804C (34.5 Å, 30.6 Å, 30.1 Å). **g**, CHX of WT and mutant NCOA7 proteins detected via an anti-Flag antibody in HEK293T cells. **h**, A schematic diagram showing the strategy for mocking human heterozygous mutations. **i**, SA-β-gal staining in WT and *NCOA7* mutant KGN cells. Scale bar, 50 μm. **j**, The percentage of SA-β-gal-positive cells was quantified in WT and *NCOA7* mutant KGN cells (*n* = 5 biological replicates for each group). *P* values (from left to right): *P = 0.0207, **P = 0.0029, ***P = 0.0004, ***P = 0.0006, ****P < 0.0001, ***P = 0.0002, **P = 0.0016, ***P = 0.0005, ****P < 0.0001. **k**, The expression levels of senescence markers *p16*^*INK4a*^ and *p21*^*Cip1*^ were determined in WT and *NCOA7* mutant KGN cells 48 h post-plasmid transfection using RT‒qPCR (*n* = 4 biological replicates for each group). Not significant (NS), *P* = 0.0555, *P = 0.0344, *P = 0.0218, *P = 0.0112, ***P = 0.0005, **P = 0.0068. **l**, E2 production was detected in the supernatants of WT and *NCOA7* mutant KGN cells after a 48-h post-transfection period followed by additional 24-h incubation in a testosterone-containing medium (*n* = 5 biological replicates for each group). ****P < 0.0001, ****P < 0.0001, ***P = 0.0001. Data in **j**–**l** are expressed as mean ± s.e.m. and compared by one-way analysis of variance (ANOVA) with Dunnett’s post hoc test. Source data

**Extended Data Fig. 1:**
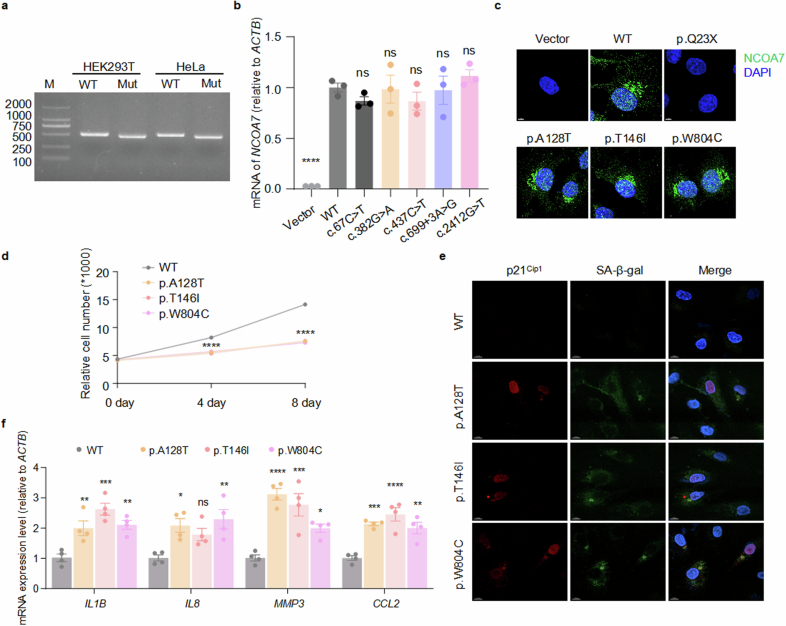
*NCOA7* mutants associated with GC senescence. (**a**) Agarose gel electrophoresis of the WT and c.699+3 A > G mutant *NCOA7* transcript. WT, wild-type; Mut, mutation. (**b**) RT‒qPCR results showing the mRNA levels of *NCOA7* in KGN cells overexpressing the WT and different mutant *NCOA7* vectors (n = 3 biological replicates per group). ****P < 0.0001, ns P > 0.9999, ns P = 0.9996, ns P = 0.6501, ns P = 0.9999, ns P = 0.9996. (**c**) Immunofluorescence images showing the localization of NCOA7 in KGN cells overexpressing the WT and different mutant *NCOA7* vectors. Scale bar: 5 μm. (**d**) Quantification of cell proliferation in *NCOA7*-KO KGN cells overexpressing the WT or mutant vectors (n = 5 biological replicates per group). ****P < 0.0001, ****P < 0.0001. (**e**) Immunofluorescence images of p21^Cip1^ and SA-β-gal in *NCOA7*-KO KGN cells overexpressing the WT or mutant vectors. Scale bar: 10 μm. (**f**) RT‒qPCR analysis of expression levels of SASP genes (*IL1B*, *IL8*, *MMP3*, *CCL2*) in *NCOA7*-KO KGN cells overexpressing the WT or mutant vectors (n = 4 biological replicates per group). **P = 0.0071, ***P = 0.0001, **P = 0.0033, *P = 0.0152, ns P = 0.0801, **P = 0.0048, ****P < 0.0001, ***P = 0.0004, *P = 0.0245, ***P = 0.0006, ****P < 0.0001, **P = 0.0018. Data in **b**, **d** and **f** are expressed as means ± s.e.m and compared by one-way ANOVA with Dunnett’s post hoc test. Source data

POI is the predominant pathological ovarian aging with a strong genetic basis^5,6^. In light of the significance of oxidative stress damage in ovarian aging, we screened our in-house whole-exome sequencing (WES) database of sporadic POI for variants in *NCOA7* (ref. ^27^), a typical member of TLDc family involved in oxidative stress response. This analysis identified five heterozygous variants in *NCOA7* (NM_001199620: c.67C>T, p.Q23X; c.382G>A, p.A128T; c.437C>T, p.T146I; c.699+3A>G; and c.2379G>T, p.W804C) across seven individuals with POI (Fig. 1a and Supplementary Table 1). After validating all the variants by Sanger sequencing, amino acid sequence alignment with NCOA7 homologs from other mammals showed that the affected residues were highly conserved across species (Fig. 1b,c). The NCOA7 protein comprises a LysM domain, a Gram domain, an estrogen receptor α (Erα) domain and a catalytic TLDc domain^28^. Structural predictions suggested that the p.Q23X nonsense variant resulted in a truncated protein lacking all four functional domains. In addition, minigene assays of RNA splicing for the c.699+3A>G predicted splice site variant revealed that this mutation introduced an alternate donor site in the middle of exon 7 that resulted in a 50-bp deletion in the open reading frame (c.650_699del), which in turn led to frameshift and premature termination codons in exon 8, and ultimately, a truncated protein (p.G217Pfs*12) (Fig. 1d and Extended Data Fig. 1a).

To assess the effects of these mutations on NCOA7 protein expression, we first transiently expressed wild-type and/or mutant *NCOA7* in a human GC line-KGN cells and compared NCOA7 protein levels by western blotting. As expected, the p.Q23X and p.G217Pfs*12 mutations resulted in undetectable or truncated NCOA7. Moreover, cells respectively expressing the three missense variants in the LysM or TLDc domains had significantly lower NCOA7 protein levels than wild-type controls (Fig. 1e), although mRNA levels remained largely unaffected (Extended Data Fig. 1b). All mutations had no significant effect on the subcellular localization of NCOA7 indicated by immunofluorescence staining (Extended Data Fig. 1c). Next, we constructed a three-dimensional structure prediction model of the NCOA7 protein, which suggested that the LysM, Gram and TLDc domains (111–161, 219–292 and 761–895 amino acids, respectively) likely form a trimer-like region. Greater distances between these three subunits are predicted in those missense variants, which destabilizes the binding interface and reduces NCOA7 protein stability; the trimer configuration is likely shattered in the two truncation variants (Fig. 1f). A further cycloheximide chase assay revealed significant decrease in the mutant NCOA7 protein levels compared to wild-type controls following 1.5-h cycloheximide treatment (Fig. 1g), suggesting that these *NCOA7* missense mutations identified in research participants with POI impair its protein stability.

Given the concomitant occurrence of oxidative stress and cellular senescence in age-related diseases^29,30^, we next examined whether the aberrant/abolished function of these variants play a role in promoting cellular senescence by checking senescence-associated markers. Plasmids expressing each of the variants with wild-type *NCOA7* (1:1 ratio) are transfected into the *NCOA7*-knockout (*NCOA7*-KO) KGN cells to mimic the heterozygous genotype of patients (Fig. 1h). Cells expressing any of the three missense variants exhibited cellular senescence phenotypes, including compromised cell proliferation, increased number of senescence-associated β-galactosidase (SA-β-gal) positive cells, and increased transcription levels of *p16*^*INK4a*^ (also known as *CDKN2A*) and *p21*^*Cip1*^ (*CDKN1A*), as well as *IL1B*, *IL8*, *MMP*3 and *CCL2*, representing a senescence-associated secretory phenotype (SASP) (Fig. 1i–k and Extended Data Fig. 1d–f). As GCs are the main source of estrogen production in the ovary, we found that these mutations were also associated with significantly reduced estradiol (E2) secretion (Fig. 1l). Together, these results demonstrate that cells carrying *NCOA7* variants are more prone to cellular senescence, which could presumably lead to premature ovarian aging.

### NCOA7 downregulation links to GC senescence in ovarian aging

**Fig. 2:**
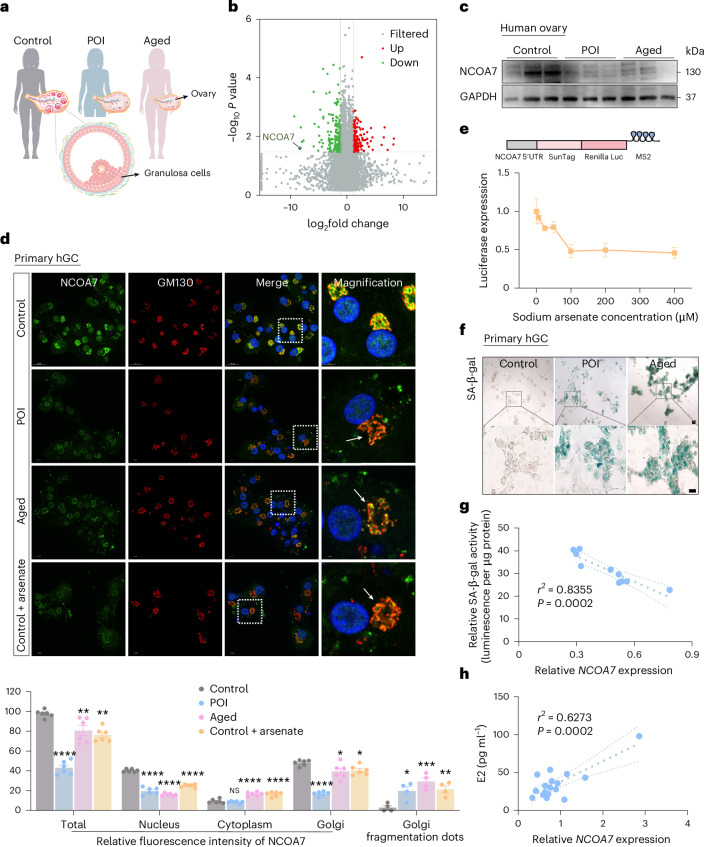
Decreased NCOA7 expression is associated with GC senescence in individuals with ovarian aging. **a**, A schematic diagram showing GCs isolated from control, POI and middle-aged (>40 years old, before menopause) research participants. **b**, Volcano plot showing DEGs (|log_2_ fold change| >1.5 and two-sided edgeR-adjusted *P* < 0.05) in GCs between control participants and participants with POI. **c**, Western blot of NCOA7 protein levels in ovarian tissues from control, POI and middle-aged participants. **d**, Immunofluorescence analysis and quantification of NCOA7 and GM130 expression in primary GCs from control, POI and middle-aged participants, and arsenate-treated GCs from control participants (*n* = 6 biological replicates for quantifying relative fluorescence intensity of NCOA7 and *n* = 4 biological replicates for quantifying Golgi fragmentation dots). For fluorescence intensity analysis of NCOA7, ‘Golgi’ refers to the region located within the Golgi apparatus and ‘cytoplasm’ denotes the cellular compartments excluding the Golgi apparatus. Relative fluorescence intensities were calculated by normalizing to the total fluorescence intensity of cells in control group. Scale bars, 20 μm or 5 μm. *P* values (from left to right): ****P < 0.0001, **P = 0.0045, **P = 0.0005, ****P < 0.0001, ****P < 0.0001, ****P < 0.0001; NS, *P* = 0.7238; ****P < 0.0001, ****P < 0.0001, ****P < 0.0001, *P = 0.0170, *P = 0.0393, *P = 0.0136, ***P = 0.0005, **P = 0.0072. **e**, A schematic diagram showing the strategy for SunTag and assessing the expression of the *NCOA7*-SunTag reporter through luciferase assays in KGN cells carrying the *NCOA7*-SunTag reporter (*n* = 5 biological replicates per group). This assessment was conducted under varying concentrations of sodium arsenate. **f**, SA-β-gal staining of GCs from control, POI and middle-aged participants. Scale bars, 20 μm or 5 μm. **g**,**h**, The correlation of *NCOA7* mRNA expression with SA-β-gal activity (**g**) (*r*^2^ = 0.8355, *P* = 0.0002, *n* = 10 biological replicates) or E2 levels (**h**) (*r*^2^ = 0.6273, *P* = 0.0002, *n* = 17 biological replicates) through Pearson correlation analysis. The dashed line indicates the 95% CI. Data in **d**,**e** are expressed as mean ± s.e.m. and compared by one-way ANOVA (with Dunnett’s post hoc test in **d**). Source data

**Extended Data Fig. 2:**
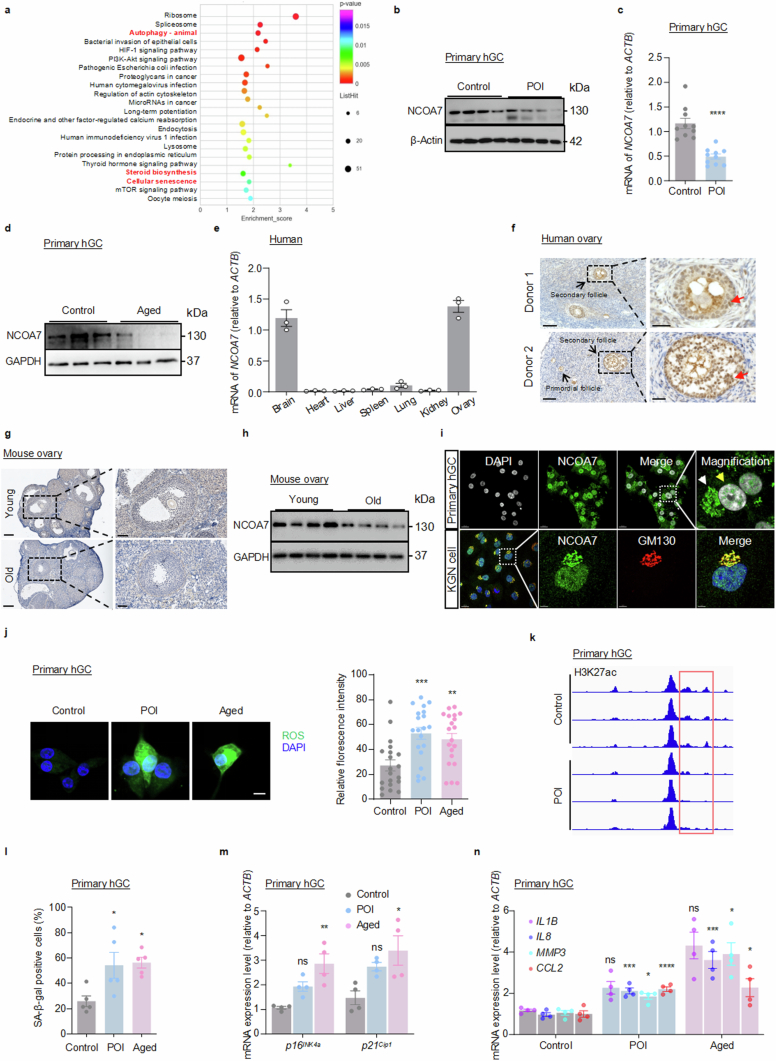
GC senescence in patients with ovarian aging is associated with reduced NCOA7 expression. (**a**) Representative KEGG pathways of DEGs in GCs between control and POI women (hypergeometric test, BH-adjusted FDR < 0.05). (**b**) Western blot of NCOA7 in primary hGCs from control and POI women. (**c**) RT‒qPCR analysis of *NCOA7* mRNA in GCs between control and POI women (n = 10 biological replicates per group). ****P < 0.0001. (**d**) Western blot of NCOA7 in GCs between control and middle-aged participants (n = 3 biological replicates per group). (**e**) RT‒qPCR analysis of *NCOA7* mRNA in various human embryonic tissues (22 weeks old) (n = 3 technical replicates per group). (**f-g**) Immunohistochemistry of NCOA7 protein in ovaries from human (**f**), as well as young (8-week-old) and old (10-month-old) mice (**g**). Red arrow: GC. Scale bar: 100 μm or 30 μm in f, 200 μm or 80 μm in g. (**h**) Western blot of NCOA7 protein levels in ovaries between young and old mice. (**i**) Immunofluorescence of NCOA7 and GM130 in primary human GCs and KGN cells. Scale bar: 20 μm or 5 μm. (**j**) Immunofluorescence and quantification of ROS in GCs from control, POI, and middle-aged patients (n = 20 views per group). Scale bar: 5 μm. ***P = 0.0005, **P = 0.0043. (**k**) CUT&Tag assay showing H3K27ac histone modification of the NCOA7 protein in GCs from control and POI women. (**l**) Quantification of SA-β-gal-positive cells in GCs from control, POI, and middle-aged patients in Fig. 2f (n = 5 biological replicates per group). *P = 0.0232, *P = 0.0156. (**m‒n**) Expression levels of *p16*^*INK4a*^ and *p21*^*Cip1*^ (**m**) and SASP genes (**n**) in GCs from control, POI, and middle-aged patients (n = 4 biological replicates per group). ns P = 0.0728, **P = 0.0015, ns P = 0.0880, *P = 0.0137 in m. ns P = 0.1611, ***P = 0.0009, *P = 0.0210, ****P < 0.0001, ns P = 0.2057, ***P = 0.0003, *P = 0.0229, *P = 0.0172 in n. Data in **c**, **e**, **j** and **l**-**n** are expressed as means ± s.e.m. Two-tailed Student’s t-test was used in **c**, and One-way ANOVA with Dunnett’s post hoc test in **j** and **l**-**n** for group comparisons. Source data

Given the protein instability resulting from *NCOA7* mutations identified in individuals with POI, we ask whether reduced expression of NCOA7 in the ovarian microenvironment is associated with ovarian aging. Ovarian GCs isolated from research participants with POI as well as advanced-aged premenopausal participants (>40 years) with physiological ovarian aging, were characterized (Fig. 2a). RNA sequencing (RNA-seq) was conducted in GCs from eight participants with POI and nine age-matched control participants undergoing assisted reproductive technology (ART) treatment. Among the differentially expressed genes (DEGs) identified in POI GCs, *NCOA7* showed a significant downregulation (Fig. 2b). Kyoto Encyclopedia of Genes and Genomes (KEGG) analysis of the DEGs showed enrichment in pathways associated with cellular senescence and autophagy, in addition to steroid biosynthesis due to characteristic estrogen depletion in GCs of individuals with POI (Extended Data Fig. 2a). Further validation by quantitative PCR with reverse transcription (RT–qPCR) and western blotting in GCs isolated from independent cohorts confirmed the decreased NCOA7 expression in GCs of participants with POI and middle-aged (>40 years old, before menopause) participants (Extended Data Fig. 2b–d and Supplementary Table 2). To characterize NCOA7 expression profile across diverse tissues, we found that NCOA7 is abundantly expressed in human ovary tissue, particularly in GCs (Extended Data Fig. 2e–f). Consistently, NCOA7 expression is markedly lower in ovarian tissues from participants with POI, middle-aged participants and aged mice compared to the control groups (Fig. 2c and Extended Data Fig. 2g,h). Thus, NCOA7 deficiency is highly involved in both pathological and physiological ovarian aging.

Subcellular localization of NCOA7 in human primary GCs or KGN cell lines by immunostaining showed that NCOA7 is present in both the cytoplasm and nucleus, which is consistent with previous studies^31,32^. Besides, ‘thread-like clusters’ of NCOA7 in the cytoplasm were found to colocalize with *cis*-Golgi marker GM130 (Extended Data Fig. 2i). Comparison of NCOA7 distribution patterns among participants with POI, middle-aged and control participants showed a decreased intensity of NCOA7 in GCs from both POI and middle-aged donors (Fig. 2d). We also observed obvious Golgi fragmentation and increased reactive oxygen species (ROS) in GCs of POI and middle-aged donors (Fig. 2d and Extended Data Fig. 2j). Similar trafficking patterns of NCOA7 were observed under oxidative stress response in primary GCs from control participants (Fig. 2d), suggesting that GCs from individuals with ovarian aging might experience chronic oxidative stress. To further investigate the potential relationship between cellular stress and NCOA7 downregulation, we used the SunTag system to image NCOA7 protein translation under oxidative stress at single-mRNA resolution^33^. In line with the above results, NCOA7 protein expression was notably inhibited in KGN cells following arsenate treatment (Fig. 2e). Furthermore, CUT&Tag analysis indicated that H3K27ac modification of *NCOA7* was significantly reduced in GCs of the POI group compared to the healthy control group (Extended Data Fig. 2k). These data suggest that NCOA7 downregulation is associated with chronic oxidative stress in ovarian GCs of individuals with ovarian aging.

We next examined the relationship between reduced *NCOA7* expression and GC senescence, and found that GCs from both individuals with POI and middle-aged participants adopt a cellular senescent state, including intensive SA-β-gal staining, elevated *p16*^*INK4a*^ and *p21*^*Cip1*^ expression, as well as *IL1B*, *IL8*, *MMP*3 and *CCL2* expression (Fig. 2f and Extended Data Fig. 2l–n). Additionally, reduced *NCOA7* mRNA levels in GCs were closely associated with increased SA-β-gal activity along with decreased E2 levels (Fig. 2g–h) (*r*^2^ = 0.8355, *P* = 0.0002; *r*^2^ = 0.6273, *P* = 0.0002, respectively). These data suggest that GCs in individuals with ovarian aging exhibit decreased NCOA7 expression and increased cellular senescence, which are closely related to oxidative stress.

## NCOA7 knockout accelerates cellular senescence and ovarian aging

**Fig. 3:**
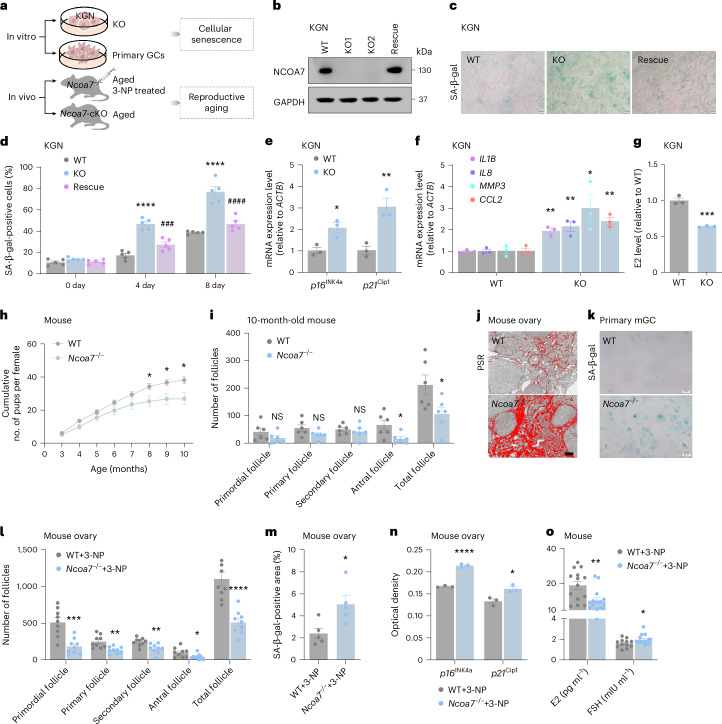
Loss of NCOA7 accelerates cellular senescence and ovarian aging. **a**, Schematic overview of in vitro and in vivo experimental designs. **b**, Validation of NCOA7 knockout efficiency in KGN cells via the CRISPR–Cas9 system. **c**,**d**, SA-β-gal staining and quantification (*n* = 5 biological replicates per group) in WT, *NCOA7*-KO and rescue KGN cells. Scale bar, 20 μm. *P* values (from left to right): ****P < 0.0001, ###*P* = 0.0006, ****P < 0.0001, ####*P* < 0.0001. **e**,**f**, RT‒qPCR analysis of *p16*^*INK4a*^ and *p21*^*Cip1*^ (**e**) and SASP genes (**f**) in WT and *NCOA7*-KO KGN cells (*n* = 3 biological replicates per group). *P = 0.0186, **P = 0.0100 (**e**). **P = 0.0021, **P = 0.0085, *P = 0.0417, **P = 0.0029 (**f**). **g**, E2 levels in WT and *NCOA7*-KO KGN cells (*n* = 3 biological replicates per group). ***P = 0.0005. **h**, Cumulative litter size in WT and *Ncoa7*^−*/*−^ female mice up to 10 months old (*n* = 10 biological replicates per genotype). *P = 0.0303, *P = 0.0226, *P = 0.0156. **i**, Ovarian follicle counts in WT and *Ncoa7*^−*/*−^ mice at 10 months old (*n* = 6 biological replicates per group). NS, *P* = 0.1812; NS, *P* = 0.1010; NS, *P* = 0.5263; *P = 0.0254; *P = 0.0354. **j**, PSR staining of ovaries in 10-month-old WT and *Ncoa7*^−*/*−^ mice. Scale bar, 100 μm. **k**, SA-β-gal staining of GCs isolated from WT and *Ncoa7*^−*/*−^ mice. Scale bar, 20 μm. **l**, Ovarian follicle counts in 3-NP-treated WT and *Ncoa7*^−*/*−^ mice (*n* = 9 biological replicates per group). ***P = 0.0003, **P = 0.0025, **P = 0.0050, *P = 0.0328, ****P < 0.0001. **m**, Quantification of the SA-β-gal-positive area in ovaries from 3-NP-treated WT and *Ncoa7*^−*/*−^ mice (*n* = 5 biological replicates per group). *P = 0.0210. **n**, Immunohistochemical quantification of p16^INK4a^ and p21^Cip1^ in ovaries from 3-NP-treated WT and *Ncoa7*^−*/*−^ mice (*n* = 3 biological replicates per group). ****P < 0.0001, *P = 0.0183. **o**, Serum levels of E2 and FSH in 3-NP-treated WT (E2: *n* = 14; FSH: *n* = 12 biological replicates) and *Ncoa7*^−*/*−^ mice (*n* = 15 biological replicates per group). **P = 0.0045, *P = 0.0407. Data in **d**–**i** and **l**–**o** are expressed as mean ± s.e.m. and compared by two-tailed Student’s *t*-test. Source data

**Extended Data Fig. 3:**
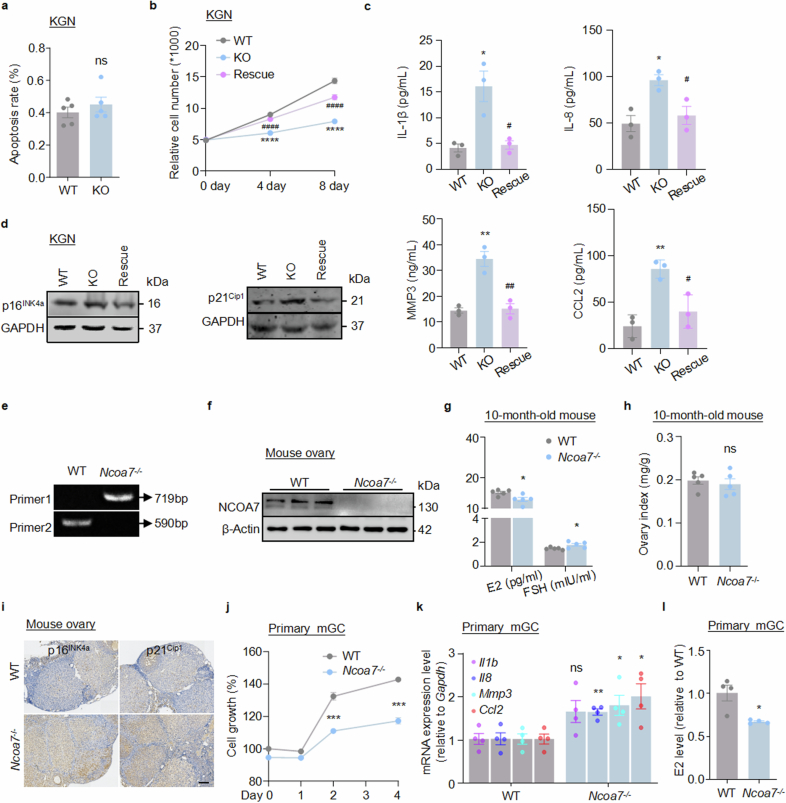
NCOA7 deficiency accelerates cellular senescence and ovarian aging. (**a**) Quantification of the apoptosis rate in WT and *NCOA7*-KO KGN cells (n = 5 biological replicates per group). ns P = 0.3981. (**b**) Quantification of cell proliferation in WT, *NCOA7*-KO, and *NCOA7*-rescued KGN cells (n = 5 biological replicates per group). Scale bar: 5 μm. The rescued group was compared with the KO group (#). ####P < 0.0001, ****P < 0.0001, ####P < 0.0001, ****P < 0.0001. (**c**) Quantification of IL-1β, IL-8, MMP3 and CCL2 in the supernatants of WT, *NCOA7*-KO, and *NCOA7*-rescued KGN cells (n = 5 biological replicates per group). The rescue group was compared with the KO group (#). *P = 0.0172, #P = 0.0206, *P = 0.0115, #P = 0.0279, **P = 0.0030, ##P = 0.0054, **P = 0.0025, #P = 0.0181. (**d**) Western blot of p16^INK4a^ and p21^Cip1^ in WT, *NCOA7*-KO, and *NCOA7*-rescued KGN cells. (**e**) PCR genotyping of WT and *Ncoa7*^−*/*−^ mice. (**f**) Western blot of ovarian tissues to confirm absence of NCOA7 protein expression in *Ncoa7*^−*/*−^ mice. (**g**) The serum levels of E2 and FSH in 10-month-old WT and *Ncoa7*^−*/*−^ mice (n = 5 biological replicates per group). *P = 0.0224, *P = 0.0412. (**h**) The ovary index in 10-month-old WT and *Ncoa7*^−*/*−^ mice (n = 4 biological replicates per group). ns P = 0.6741. (**i**) Immunohistochemistry analysis of p16^INK4a^ and p21^Cip1^ in ovaries of WT and *Ncoa7*^−*/*−^ mice (n = 3 biological replicates per group). Scale bar: 100 μm. *P = 0.0135, ***P = 0.0006. (**j-k**) Quantification of cell proliferation (n = 3 biological replicates per group) (**j**) and expression levels of SASP genes determined via RT‒qPCR (n = 4 biological replicates per group) (**k**) in GCs from WT and *Ncoa7*^−*/*−^ mice. ***P = 0.0010, ***P = 0.0003 in (r). ns P = 0.0661, **P = 0.0072, *P = 0.0241, *P = 0.0196 in (s). (**l**) Quantification of E2 production in the supernatants of GCs isolated from WT and *Ncoa7*^−*/*−^ mice (n = 4 biological replicates per group). *P = 0.0106. Data in **a**-**c**, **g**-**h**, and **j**-**l** are expressed as means ± s.e.m, and compared by two-tailed Student’s t-test. Source data

To better understand how NCOA7 deficiency contributes to GC senescence and ovarian aging, we generated *NCOA7*-KO KGN cells through CRISPR–Cas9-mediated genome editing and verified NCOA7 ablation by western blotting (Fig. 3a,b). No significant difference in cell apoptosis was observed between *NCOA7*-KO and wild-type KGN cells (Extended Data Fig. 3a). Growth curves showed increased senescence in successive passages of *NCOA7*-KO cells, mirrored by higher percentages of SA-β-gal-positive cells (Fig. 3c–d and Extended Data Fig. 3b). Furthermore, senescence markers (p16^INK4a^ and p21^Cip1^) and SASP (IL-1β, IL-8, MMP3 and CCL2) were upregulated in *NCOA7*-KO cells compared to wild-type controls (Fig. 3e,f and Extended Data Fig. 3c,d). The *NCOA7*-KO cells also exhibited compromised capacity in E2 production, indicating intrinsically impaired steroidogenic function (Fig. 3g). To confirm that the above effects were specific to *NCOA7*, we expressed wild-type *NCOA7* plasmids in the *NCOA7*-KO clones, which resulted in increased cell growth and decreased senescence marker expression (Fig. 3b–d and Extended Data Fig. 3b–d), indicating substantial rescue of the senescence phenotype. These cumulative results demonstrated that NCOA7 ablation could promote cell senescence in vitro.

**Extended Data Fig. 4:**
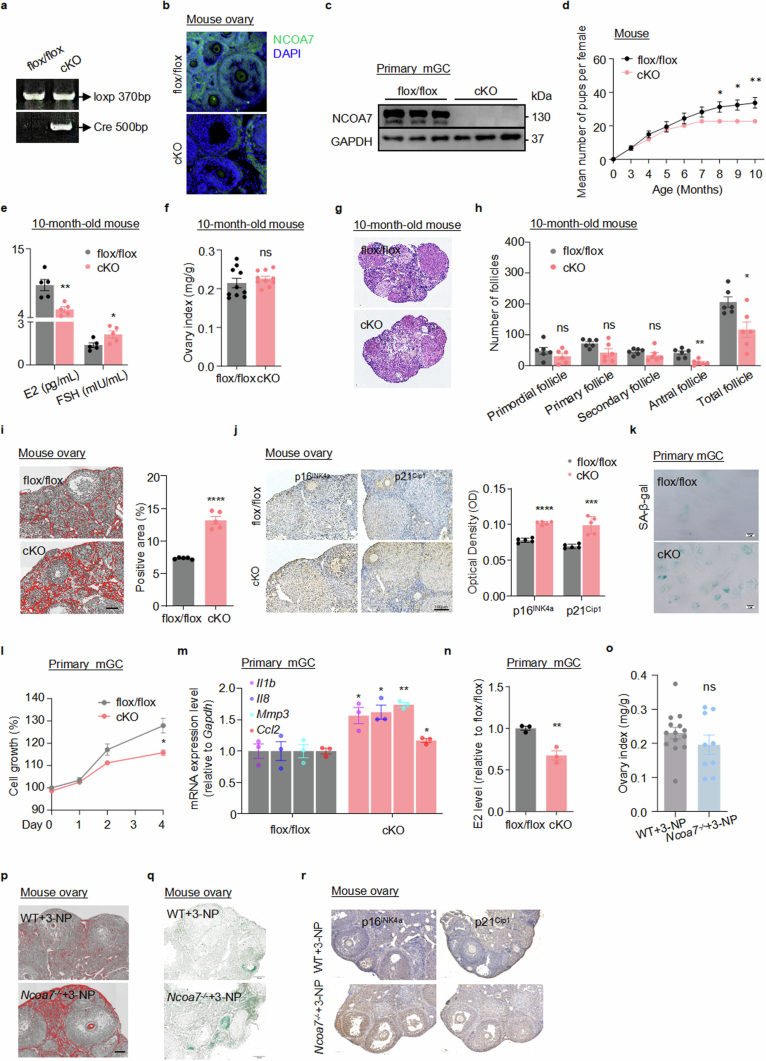
NCOA7 deficiency accelerates ovarian aging in mice. (**a**) PCR genotyping of flox/flox and *Ncoa7-*cKO female mice. (**b**) Immunofluorescence of NCOA7 in ovaries of flox/flox and *Ncoa7-*cKO mice. Scale bar: 50 μm. (**c**) Western blot confirmed NCOA7 absence in GCs of *Ncoa7-*cKO mice. (**d**) Cumulative litter size in flox/flox and *Ncoa7-*cKO female mice up to 10-month-old (n = 7 biological replicates per genotype). *P = 0.0211, *P = 0.0104, **P = 0.0063. (**e**) Serum E2 and FSH levels in 10-month-old flox/flox and *Ncoa7-*cKO mice (n = 5 biological replicates per group). **P = 0.0042, *P = 0.0356. (**f**) Ovary index in 10-month-old flox/flox and *Ncoa7-*cKO mice (n = 10 biological replicates per group). ns P = 0.3992. (**g**) HE staining of ovarian sections from 10-month-old flox/flox and *Ncoa7-*cKO mice. Scale bar: 300 μm. (**h**) Ovarian follicle counts in flox/flox and *Ncoa7-*cKO mice at 10-month-old (n = 6 biological replicates per group). ns P = 0.2509, ns P = 0.0514, ns P = 0.2570, **P = 0.0010, *P = 0.0151. (**i**) PSR staining and quantification in 10-month-old flox/flox and *Ncoa7-*cKO mice. Scale bar: 100 μm. ****P < 0.0001. (**j**) Immunohistochemistry of p16^INK4a^ and p21^Cip1^ in ovaries of flox/flox and *Ncoa7-*cKO mice. Scale bar: 100 μm. ****P < 0.0001, ***P = 0.0007. (**k-m**) SA-β-gal staining (**k**), cell proliferation (n = 3 biological replicates per group) (**l**) and SASP genes expression (n = 3 biological replicates per group) (**m**) in GCs from flox/flox and *Ncoa7-*cKO mice. Scale bar: 20 μm. *P = 0.0258 in l, *P = 0.0301, *P = 0.0299, **P = 0.0027, *P = 0.0366 in m. (**n**) E2 levels in GCs isolated from flox/flox and *Ncoa7-*cKO mice (n = 3 biological replicates per group), *P = 0.0058. (**o**) Ovary index of WT (n = 14 biological replicates) and *Ncoa7*^−*/*−^ mice (n = 9 biological replicates) treated with 3-NP. Scale bar: 100 μm. ns P = 0.2794. (**p-r**) PSR staining (**p**), SA-β-gal staining (**q**), and immunohistochemistry of p16^INK4a^ and p21^Cip1^ (**r**) in ovaries from WT and *Ncoa7*^−*/*−^ mice treated with 3-NP. Scale bars: 100 μm. Data in **d**-**f**, **h**-**j**, and **l**-**o** are expressed as means ± s.e.m, and compared by two-tailed Student’s t-test. Source data

To test whether NCOA7 deficiency could drive ovarian aging in vivo, we generated *Ncoa7* knockout mice (*Ncoa7*^−*/*−^) by CRISPR–Cas9 genome editing. After confirming the homozygous *Ncoa7*-KO genotype and loss of protein expression in ovaries by western blotting (Extended Data Fig. 3e,f), we observed that *Ncoa7*^−*/*−^ female mice showed age-related fertility decline, with decreased litter size from 8 months of age onward, compared to wild-type controls (Fig. 3h). Increased follicle-stimulating hormone (FSH) levels and decreased E2 levels were found in the serum of *Ncoa7*^−*/*−^ mice at 10 months old (Extended Data Fig. 3g). Moreover, *Ncoa7*^−*/*−^ mice had a significantly reduced number of ovarian follicles, especially the antral follicles (Fig. 3i and Extended Data Fig. 3h). Subsequent Picro Sirius red (PSR) assays revealed a prominent network of intensely stained collagen fibers throughout the ovarian stroma and albuginea, indicating increased ovarian fibrosis in *Ncoa7*^−*/*−^ mice (Fig. 3j). Elevated p16^INK4a^ and p21^Cip1^ levels indicated an enhanced ovarian aging phenotype in aged *Ncoa7*^−*/*−^ mice compared to wild-type controls (Extended Data Fig. 3i). Consistent with the above in vitro data, ovarian GCs from *Ncoa7*^−*/*−^ mice showed increased cellular senescence and reduced E2 secretion compared to GCs from wild-type mice (Fig. 3k and Extended Data Fig. 3j–l). To further dissect the role of GC-specific NCOA7 in ovarian aging, we conditionally deleted *Ncoa7* in ovarian GCs by using a *Foxl2*^*cre*^-mediated recombination strategy (*Foxl2*^*cre*^;*Ncoa7*^*flox/flox*^, *Ncoa7*-cKO). Genotyping assay, immunofluorescence of ovarian sections and western blot analysis of isolated GCs confirmed the absence of NCOA7 expression specifically in GCs within the ovaries of *Ncoa7*-cKO mice (Extended Data Fig. 4a–c). In line with the phenotype of global knockout mice, the *Ncoa7*-cKO mice also showed age-related fertility decline, elevated FSH levels and decreased E2 levels in the serum at 10 months old, reduced follicle numbers, increased ovarian fibrosis, enhanced ovarian aging phenotype and cellular senescence in ovarian GCs (Extended Data Fig. 4d–n). Together, these results suggested that loss of NCOA7 promotes cellular senescence and consequently accelerates ovarian aging, similar to the ovarian aging phenotype of human patients.

To further examine whether NCOA7 deficiency accelerates ovarian aging under oxidative stress, we induced oxidative stress damage in 6-week-old wild-type and *Ncoa7*^−*/*−^ mice by administering 3-nitropropionic acid (3-NP) intraperitoneally for 3 weeks (Fig. 3a). While ovary size was marginally reduced in *Ncoa7*^−*/*−^ mice, a marked decrease in follicle counts was found at different developmental stages (Fig. 3l and Extended Data Fig. 4o). Consistently, *Ncoa7*^−*/*−^ ovaries treated with 3-NP displayed enhanced fibrosis and cellular senescence (SA-β-gal, p16^INK4a^ and p21^Cip1^) (Fig. 3m,n and Extended Data Fig. 4p–r). In addition, levels of FSH were increased while E2 levels were decreased in serum of 3-NP treated *Ncoa7*^−*/*−^ mice (Fig. 3o). Overall, these data indicate that NCOA7 deficiency in ovaries increases sensitivity to oxidative stress, promoting cell senescence and consequently ovarian aging.

## NCOA7 promotes LLPS via LysM–NTF2L domain interaction with G3BP1

**Fig. 4:**
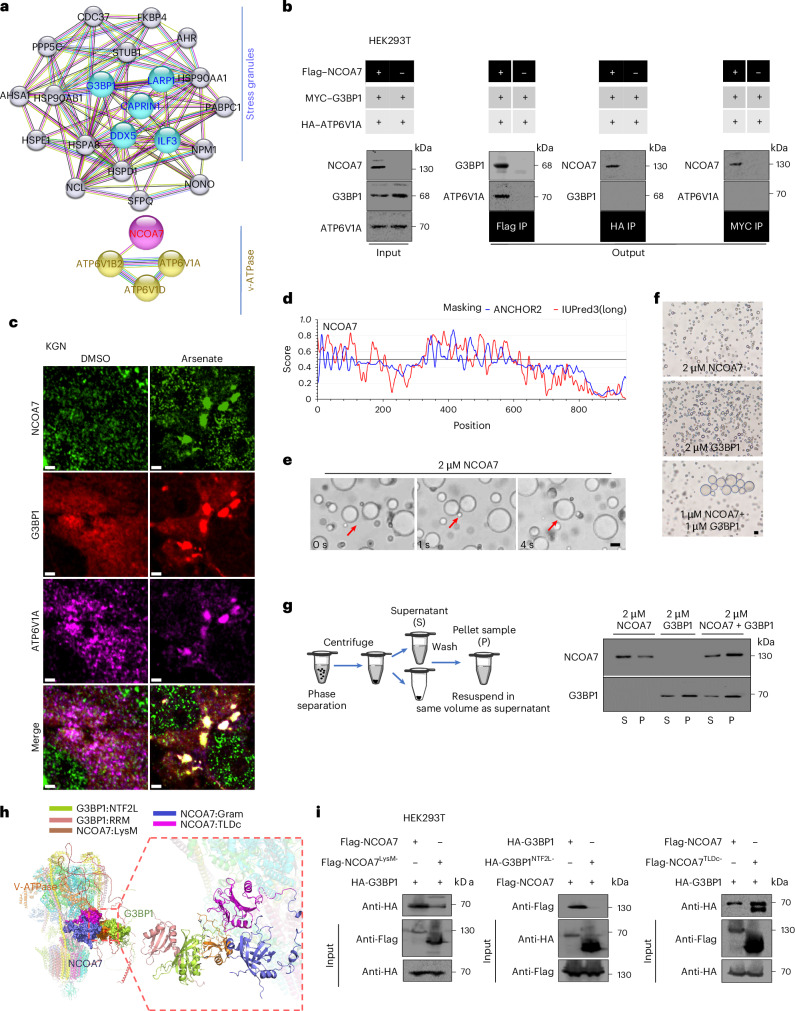
Interaction of NCOA7 with G3BP1 via the LysM and NTF2L domains enhances phase separation in vitro. **a**, Protein–protein interaction network showing the NCOA7 interactome, including components of SGs and V-ATPase identified by IP–MS. **b**, Co-IP assays showing interactions of NCOA7, G3BP1 and ATP6V1A in HEK293T cells. **c**, Immunofluorescence showing colocalization of NCOA7, G3BP1 and ATP6V1A in KGN cells treated with arsenate or DMSO. Scale bar, 2 μm. **d**, Prediction analysis of the intrinsically disordered region of NCOA7 by IUPred (http://iupred.enzim.hu/). **e**, Time-lapse images of NCOA7 protein droplets that undergo rapid fusion (arrows). The time point of the first image is defined as 0 s. Scale bar, 20 μm. **f**, Differential interference contrast images showing droplets of NCOA7 (2 μM), G3BP1 (2 μM) and a mixture of NCOA7 (1 μM) and G3BP1 (1 μM). Scale bar, 20 μm. **g**, A schematic flowchart showing the protein isolation process and western blot detection of NCOA7 and G3BP1 levels in the corresponding supernatant (S) and pellet (P). **h**, Predicted three-dimensional structure showing the binding positions and interaction mode analysis of the NCOA7–G3BP1–V-ATPase complex. **i**, Co-IP validation of the interaction between NCOA7 and G3BP1 with different domain deletions. Source data

**Extended Data Fig. 5:**
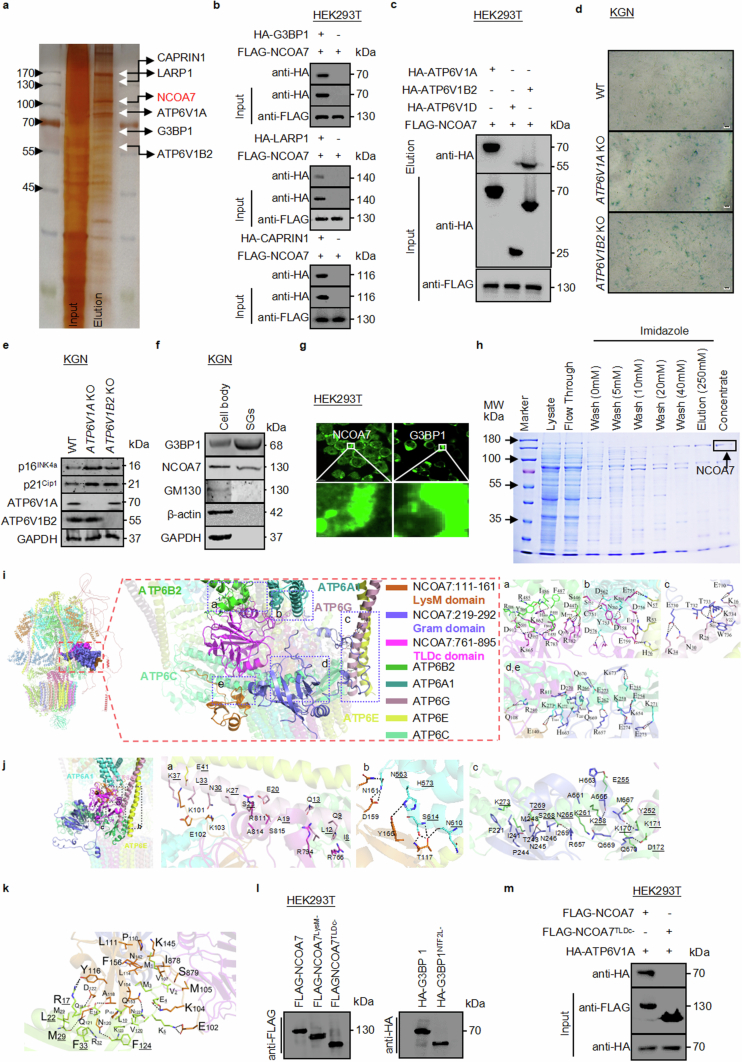
NCOA7 interacts with SG components. (**a**) A FLAG-pulldown assay to identify NCOA7-binding proteins in KGN cells. Specific bands were identified via MS. (**b-c**) Co-IP validation of the binding of G3BP1, LARP1, CAPRIN1 (**b**) and ATP6V1 subunits (ATP6V1A, ATP6V1B2 and ATP6V1D) (**c**) to NCOA7 in HEK293T cells. (**d-e**) SA-β-gal staining (**d**) and western blot of senescence markers (p16^INK4a^ and p21^Cip1^) (**e**) in WT, *ATP6V1A*- and *ATP6V1B2*-KO KGN cells. Scale bar: 20 μm. (**f**) Western blot of G3BP1 and NCOA7 within the isolated SG fractions. GM130 as a negative control. (**g**) Immunofluorescence images of HEK293T cells overexpressing *NCOA7* or *G3BP1* plasmids. Scale bar: 20 μm or 2 μm. (**h**) SDS‒PAGE analysis of samples collected during the purification process to confirm the validity of the purification. (**i-j**) Docking model showing the binding positions, interaction mode analysis and amino acid analysis at the binding surface of five subunits (ATP6A1, ATP6B2, ATP6C, ATP6E, and ATP6G) of V-ATPase and WT NCOA7 (**i**) or mutant NCOA7 (p.A128T) (**j**). (**k**) Amino acid analysis of the binding surface of G3BP1 and NCOA7. (**l**) Western blot of FLAG-NCOA7 (full-length or truncated versions) or HA-G3BPI (full-length or truncated version) overexpressed in HEK293T cells. The shown immunoblot was detected via an anti-Flag antibody or anti-HA antibody. (**m**) Co-IP validation of the interaction domains of NCOA7 and ATP6V1A. Source data

To define the molecular mechanism through which NCOA7 deficiency mediates stress-related cellular senescence, we first sought to identify potentially unrecognized interaction partners of NCOA7 through co-immunoprecipitation (co-IP) coupled with mass spectrometry (MS) in HEK293T cells overexpressing Flag–NCOA7 (Extended Data Fig. 5a). This analysis uncovered a notable enrichment with several known components of SGs, typically formed in response to stress conditions, in addition to the expected overrepresentation of V-ATPase complex proteins (Fig. 4a). Co-IP assays corroborated that NCOA7 interacts with several specific, essential SG constituents, including G3BP1, CAPRIN1 and LARP1 (refs. ^34,35^) and verified its interactions with ATP6V1A and ATP6V1B2 (Extended Data Fig. 5b,c). Notably, G3BP1 and ATP6V1A could interact only in the presence of NCOA7 (Fig. 4b), suggesting that NCOA7 likely functioned as an adaptor to link V-ATPase and SGs. Immunofluorescent detection of NCOA7 subcellular localization patterns in association with SGs under stress conditions intriguingly showed that both NCOA7 and ATP6V1A accumulate in cytoplasmic SGs, whereas both proteins are diffused throughout the nucleus, Golgi and cytoplasm in unstressed KGN cells (Fig. 4c). Consistent with the previous report^36,37^, ATP6V1A or ATP6V1B2 could interact with NCOA7 and their deficiency in KGN cells resulted in enhanced cellular senescence phenotypes, including increased expression of SA-β-gal, p16^INK4a^ and p21^Cip1^ (Extended Data Fig. 5d,e). We next sought to explore the role of NCOA7–G3BP1 interaction in SGs. We isolated the SG components from arsenate-stimulated KGN cells and verified the presence of NCOA7 and G3BP1, but not GM130 (a negative control), in the SGs (Extended Data Fig. 5f).

SG assemble through liquid–liquid phase separation (LLPS) arising from G3BP1 cored interactions^34^. To characterize the possible role of NCOA7 in LLPS, we first conducted predictive analysis with IUPred (http://iupred.enzim.hu/) to search for intrinsically disordered regions in the NCOA7 protein structure (Fig. 4d). Next, we overexpressed NCOA7 and observed that NCOA7 accumulated into numerous punctate structures similar to those in cells overexpressing G3BP1 (Extended Data Fig. 5g). Subsequent bright-field microscopy of NCOA7 purified from *Escherichia* *coli* could form spherical droplets at concentrations of 2 µM in low-salt buffer (150 mM NaCl), suggesting that NCOA7 could undergo phase separation in vitro (Fig. 4e–g and Extended Data Fig. 5h). We also found that under these conditions, 2 µM G3BP1 could form more abundant droplets than NCOA7, and that combining NCOA7 with G3BP1 resulted in formation of larger droplets than either protein alone, and that LLPS occurred at a lower concentration (1 µM) of each protein (Fig. 4f). These results supported that NCOA7 and G3BP1 mutually promote LLPS in SG formation.

Molecular dynamics simulations to predict the conformational states of a putative NCOA7–G3BP1–V-ATPase complex suggested that the interior region at the bottom of the V-ATPase provided an accessible binding cavity that could potentially accommodate the predicted trimer-like region of NCOA7 (Fig. 4h and Extended Data Fig. 5i). Consistent with previous reports of its likely binding interface^37^, five subunits (ATP6A1, ATP6B2, ATP6C, ATP6E and ATP6G) were predicted to directly interact with NCOA7, with polar interactions, salt bridges and hydrophobic interactions potentially contributing to complex stabilization (Extended Data Fig. 5i and Supplementary Table 3), whereas the p.A128T variant located in LysM domain identified in our individuals with POI, resulted in a conformationally looser trimer-like region with weaker interactions at the binding interface (Extended Data Fig. 5j). Subsequent modeling to investigate how G3BP1 might interact with the NCOA7–V-ATPase complex suggested that the NTF2L domain of G3BP1 could potentially bind with the LysM domain (219–292 amino acids) of NCOA7 (Fig. 4h and Extended Data Fig. 5k). Validation of the predicted binding orientations by immunoprecipitation experiments with ectopically expressed NCOA7 variants lacking their respective LysM (*NCOA7*^LysM-truncated^) or TLDc domains (*NCOA7*^TLDc-truncated^) and G3BP1, as well as ATP6V1A plasmids, indicated that the TLDc domain was required for interaction with V-ATPase but not G3BP1; whereas the LysM domain was required for NCOA7 interaction with G3BP1 (Fig. 4i and Extended Data Fig. 5l,m). Alternatively, truncation of the NTF2L domain in G3BP1 (*G3BP1*^NTF2L-truncated^) completely abolished binding of G3BP1 to NCOA7 (Fig. 4i), which further supported our in silico predictions.

### NCOA7 promotes SG clearance via autophagy post-stress in KGN cells

**Fig. 5:**
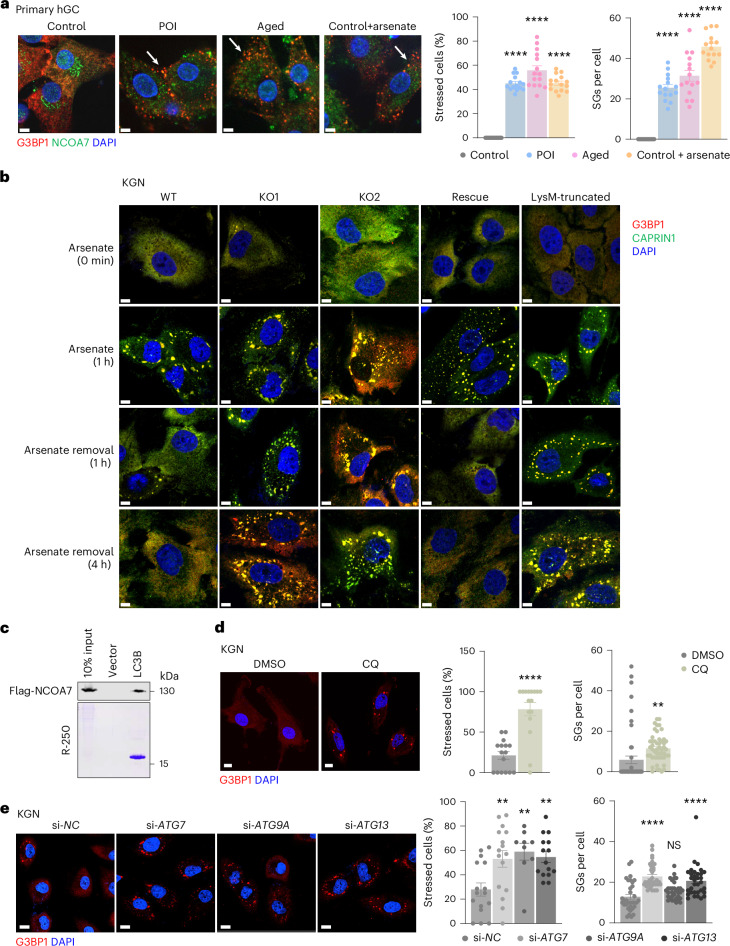
NCOA7 promotes SG clearance via autophagy after stress removal. **a**, Immunofluorescence and quantification of SGs in GCs from control, POI and middle-aged participants (*n* = 15 views per group). Scale bar, 5 μm. *P* values (from left to right): ****P < 0.0001, ****P < 0.0001, ****P < 0.0001, ****P < 0.0001, ****P < 0.0001, ****P < 0.0001. **b**, Immunofluorescence of SGs (marked by G3BP1 and CAPRIN1) in WT, *NCOA7*-KO, *NCOA7*-rescued and NCOA7 domain-truncated KGN cells after treatment with arsenate and subsequent recovery for the indicated time periods. Scale bar, 5 μm. **c**, A His pulldown assay of NCOA7–Flag from arsenate-treated cells using His–LC3 proteins. **d**, Immunofluorescence and quantification of SGs in KGN cells treated with DMSO or chloroquine (CQ) (*n* = 16 views in quantification of stressed cells; *n* = 50 views per group in quantification of SGs per cell). Scale bar, 10 μm. ****P < 0.0001, **P = 0.0094. **e**, Immunofluorescence and quantification of SGs in *ATG7-*, *ATG9A-* and *ATG13-*knockdown KGN cells (*n* = 16, 16, 9 and 15 views, respectively in quantification of stressed cells; *n* = 33 views per group in quantification of SGs per cell). Scale bar, 10 μm. **P = 0.0065, **P = 0.0043, **P = 0.0045, ****P < 0.0001; NS, *P* = 0.1502; ****P < 0.0001. Data in **a**,**d**,**e** are expressed as mean ± s.e.m. and a one-way ANOVA with Dunnett’s post hoc test (**a**,**e**) and a two-tailed Student’s *t*-test (**d**) were used for group comparisons. Source data

**Extended Data Fig. 6:**
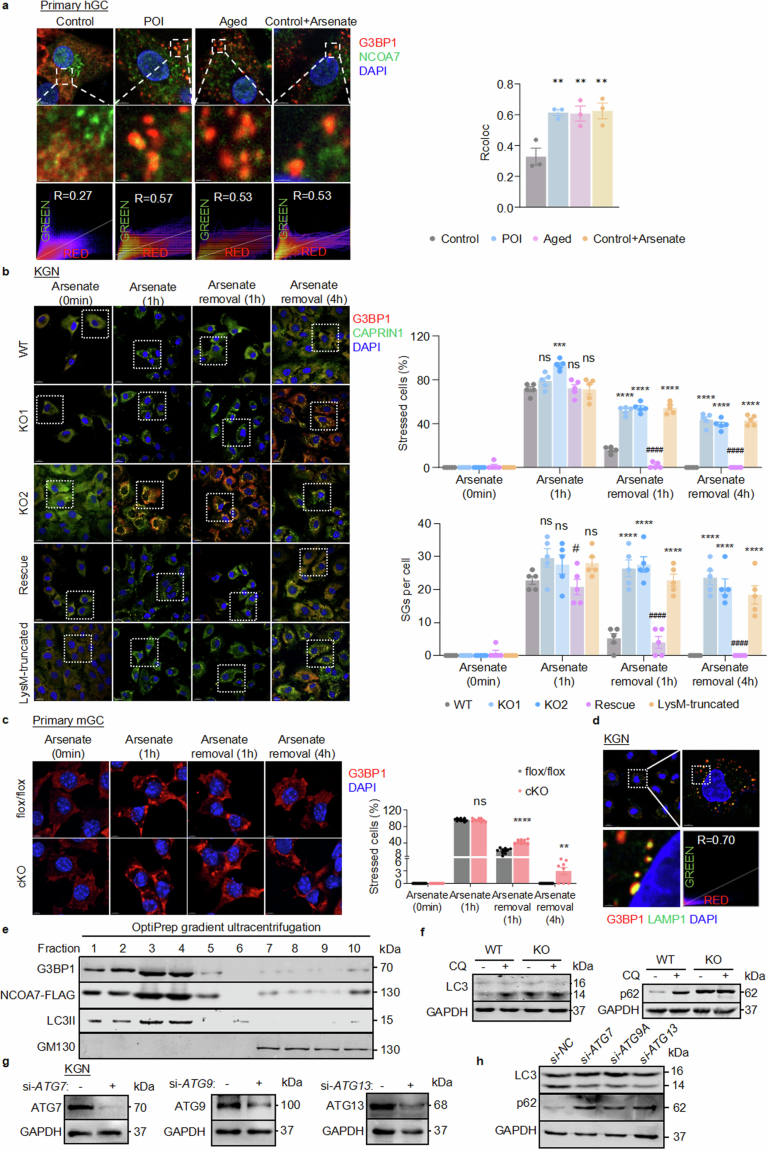
NCOA7 involved in autophagic clearance of SGs. (**a**) Immunofluorescence of colocalization and Pearson’s correlation coefficient of G3BP1 and NCOA7 in GCs from control, POI, and middle-aged participants (n = 3 biological replicates per group). Scale bar: 5 μm or 1 μm. **P = 0.0056, **P = 0.0065, **P = 0.0045. (**b**) Immunofluorescence and quantification of SGs (marked by G3BP1 and CAPRIN1) in WT, *NCOA7*-KO, *NCOA7*-rescued, and *NCOA7* LysM domain-truncated KGN cells (n = 5 biological replicates per group) under arsenate treatment and removal. Scale bar: 20 μm. The rescue group was compared with the KO1 group (#). ns P = 0.3037, ***P = 0.0003, ns P = 0.5806, ns P > 0.9999, ****P < 0.0001, ****P < 0.0001, ####P < 0.0001, ****P < 0.0001, ****P < 0.0001, ****P < 0.0001, ####P < 0.0001, ****P < 0.0001, ns P = 0.1256, ns P = 0.3448, #P = 0.0417, ns P = 0.2865, ****P < 0.0001, ****P < 0.0001, ####P < 0.0001, ****P < 0.0001, ****P < 0.0001, ****P < 0.0001, ####P < 0.0001, ****P < 0.0001. (**c**) Immunofluorescence and quantification of SGs in GCs from flox/flox and *Ncoa7*-cKO mice (n = 10 views per group). Scale bar: 5 μm. ns P = 0.6844, ****P < 0.0001, **P = 0.0025. (**d**) Immunofluorescence images of colocalization and Pearson’s correlation coefficient of G3BP1 and LAMP1 in KGN cells under arsenate treatment. Scale bar: 20 μm, 5 μm or 1 μm. (**e**) Western blot of G3BP1, NCOA7–FLAG and LC3II protein levels in various autophagosome fractions from KGN cells transfected with *NCOA7–FlagLAG* vector. GM130 as a negative control. (**f**) Western blot of LC3 and p62 protein levels in WT and *NCOA7*-KO KGN cells treated with or without CQ. (**g**) Western blot verifying the knockdown efficiency of *ATG7*, *ATG9* and *ATG13* in KGN cells. ***P = 0.0004, ***P = 0.0007, ***P = 0.0007. (**h**) Western blot of LC3 and p62 protein levels in *ATG7-*, *ATG9A-*, and *ATG13-*knockdown KGN cells. Data in **a**-**c** are expressed as means ± s.e.m. One-way ANOVA with Dunnett’s post hoc test was used in **a**-**b**, and two-tailed Student’s t-test in **c** for group comparisons. Source data

To better understand the role of SG metabolism in GC senescence, we compared SG accumulation in ovarian GCs isolated from control participants with those from participants with POI and middle-aged participants. Immunofluorescence staining showed greater numbers of G3BP1-positive puncta in GCs of individuals with POI and middle-aged donors compared to those of healthy control participants, confirming that SG accumulation was enhanced in individuals with ovarian aging. Correspondingly, we found that, in control GCs, arsenate-induced stress resulted in a similar phenotype to that of GCs from individuals with ovarian aging (Fig. 5a and Extended Data Fig. 6a), suggesting that aberrant accumulation of SGs in patients might be related to GC senescence and ovarian aging.

We then investigated the function of NCOA7 in SG formation and clearance kinetics. Quantitative analysis of the SG marker proteins, G3BP1 and CAPRIN1, in arsenate-treated wild-type and *NCOA7*-KO granule-positive KGN cells showed no difference in SG abundance, indicating that NCOA7 did not participate in SG formation under arsenate stress. Furthermore, upon removal of arsenate, SGs were almost undetectable in wild-type controls but remained consistently abundant in two independent *NCOA7*-KO KGN clones. This SG accumulation could be rescued by re-expression of wild-type *NCOA7* but not the N-terminal-truncation variant (*NCOA7*^LysM-truncated^) (Fig. 5b and Extended Data Fig. 6b). These results indicated that although NCOA7 did not have any clear role in arsenate-induced SG formation, it was necessary for SG clearance in the absence of stress stimuli. Similar results were obtained in GCs from *Ncoa7*-cKO mice (Extended Data Fig. 6c), further supporting that an intact NCOA7 configuration is necessary for SG clearance after stress removal.

Although previous reports found evidence supporting that SGs could be removed by autophagy, these results have remained controversial^38,39^. We therefore checked whether autophagy mediates NCOA7-dependent SG clearance. Co-IP assays corroborated that NCOA7 indeed associated with LC3, and immunofluorescence demonstrated significant colocalization of G3BP1 and lysosomes in KGN cells (Fig. 5c and Extended Data Fig. 6d). Moreover, we isolated autophagosomes from stressed cells, and subsequent western blotting analysis verified that LC3II-positive autophagosomes contain G3BP1 and NCOA7 (Extended Data Fig. 6e), suggesting that a key role of NCOA7 in granulophagy. Pretreatment with the autophagy inhibitor chloroquine resulted in impaired autophagic flux and increased SG accumulation in KGN cells at 1 h after arsenate removal (Fig. 5d and Extended Data Fig. 6f). Aligning well with these results, knockdown of *ATG7*, *ATG9A* or *ATG13* to inhibit autophagy in KGN cells also inhibited SG elimination (Fig. 5e and Extended Data Fig. 6g–h), further supporting that autophagy is involved in SG clearance in KGN cells. Collectively, these data suggest that NCOA7 acts as a selective autophagy adaptor for SG degradation.

### Autophagy mitigates NCOA7-defective GC senescence and ovarian aging

**Fig. 6:**
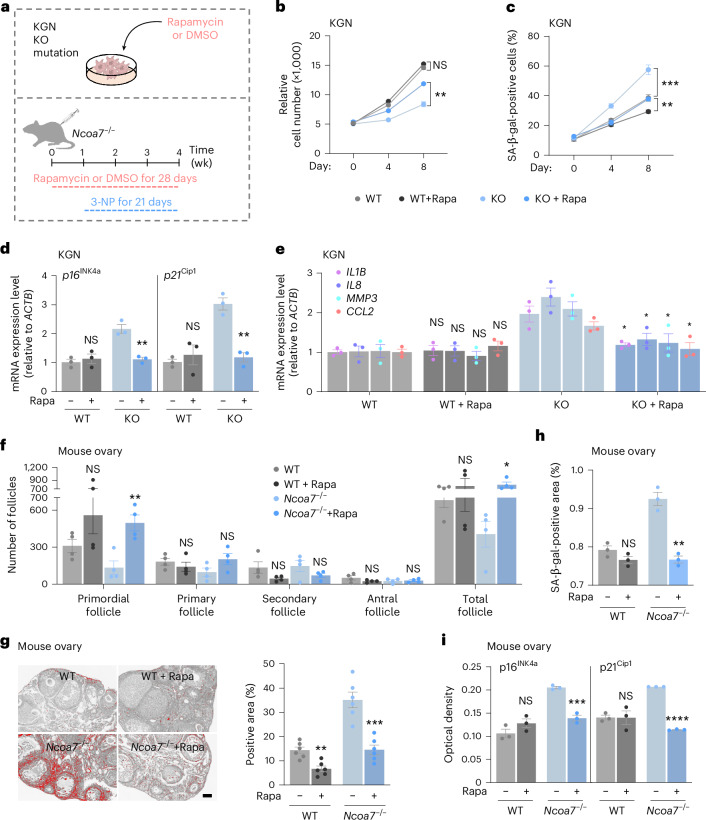
Increasing autophagy alleviates NCOA7 deficiency-related GC senescence and ovarian aging. **a**, Schematic diagram of in vitro (*NCOA7*-KO or mutant KGN cells) and in vivo (*Ncoa7*^−*/*−^ mice) experimental designs. **b**,**c**, Quantification of cell proliferation (**b**) (*n* = 3 biological replicates per group) and SA-β-gal-positive cells (**c**) (*n* = 5 biological replicates per group) in WT and *NCOA7*-KO KGN cells treated with DMSO or rapamycin (Rapa). NS, *P* = 0.1641; **P = 0.0011 (**b**). ***P = 0.0004, **P = 0.0067 (**c**). **d**,**e**, Quantification of senescence marker genes (**d**) (*n* = 3 biological replicates per group) and SASP genes (**e**) (*n* = 3 biological replicates per group) in WT and *NCOA7*-KO KGN cells treated with DMSO or rapamycin. NS, *P* = 0.5614; **P = 0.0031;, NS, *P* = 0.5241; **P = 0.0022 (**d**). NS, *P* = 0.7822; NS, *P* = 0.9464; NS, *P* = 0.5710; NS, *P* = 0.3430; *P = 0.0198; *P = 0.0176; *P = 0.0412; *P = 0.0375 (**e**). **f**, Ovarian follicle counts in 3-NP-treated WT and *Ncoa7*^−*/*−^ mice with DMSO or rapamycin pre-treatments (*n* = 4 biological replicates per group). NS, *P* = 0.1730; **P = 0.0054; NS, *P* = 0.4121; NS, *P* = 0.1052; NS, *P* = 0.1076; NS, *P* = 0.1709; NS, *P* = 0.1653; NS, *P* = 0.8376; NS, *P* = 0.6586; *P = 0.0176. **g**, PSR staining and quantification of the PSR-positive area in 3-NP-treated WT and *Ncoa7*^−*/*−^ mice with DMSO or rapamycin pre-treatments (*n* = 6 biological replicates per group). Scale bar, 100 μm. **P = 0.0013, ***P = 0.0003. **h**, Quantification of the SA-β-gal-positive area in ovaries from 3-NP-treated WT and *Ncoa7*^−*/*−^ mice with DMSO or rapamycin pre-treatments (*n* = 3 biological replicates per group). NS, *P* = 0.1352; **P = 0.0013. **i**, Quantification of optical density (OD) of immunohistochemical staining for senescence markers (p16^INK4a^ and p21^Cip1^) in ovaries from 3-NP-treated WT and *Ncoa7*^−*/*−^ mice with DMSO or rapamycin pretreatments (*n* = 3 biological replicates per group). NS, *P* = 0.1750; ***P = 0.0006; NS, *P* = 0.9798, ****P < 0.0001. Data in **b**–**i** are expressed as mean ± s.e.m. and compared by two-tailed Student’s *t*-test. Source data

**Extended Data Fig. 7:**
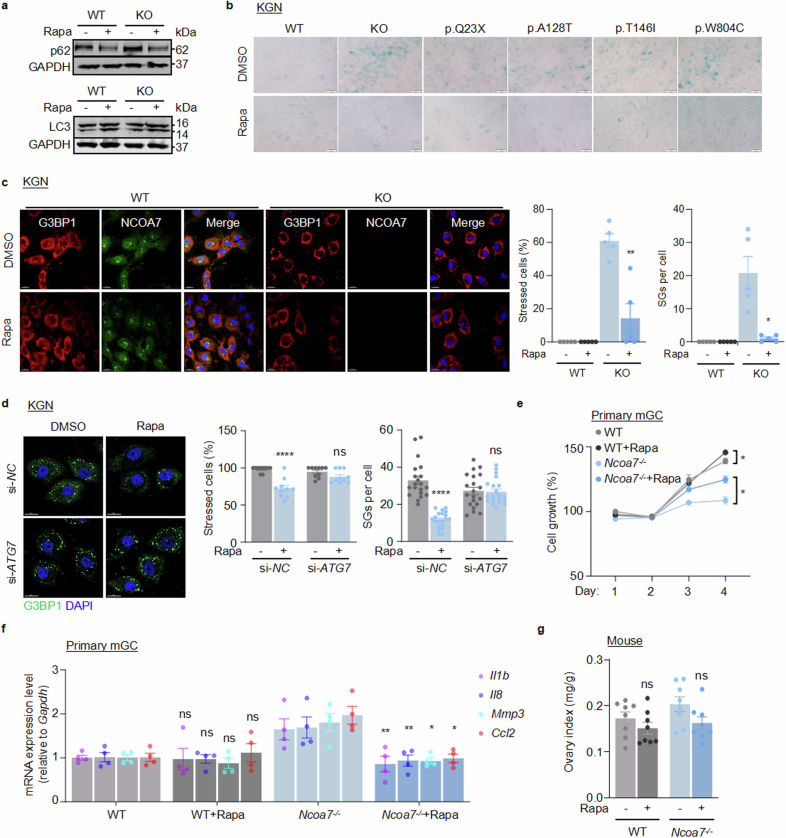
Increased autophagy alleviates NCOA7 deficiency-related GC senescence and ovarian aging both *in vitro* and *in vivo.* (**a**) Western blot of LC3 and p62 protein levels in WT and *NCOA7*-KO KGN cells treated with DMSO or rapamycin. (**b**) SA-β-gal staining of WT, *NCOA7*-KO, and *NCOA7*-mutant KGN cells. Scale bar: 50 μm. (**c**) Immunofluorescence and quantitative analysis of G3BP1 and NCOA7 in WT and *NCOA7*-KO KGN cells (n = 5 biological replicates per group) treated with DMSO or rapamycin. Scale bar: 20 μm. **P = 0.0013, *P = 0.0173. (**d**) Immunofluorescence and quantitative analysis of SGs in *ATG7*-knockdown KGN cells (n = 10 views in quantification of stressed cells; n = 20 views per group in quantification of SGs per cell). Scale bar: 15 μm. ****P < 0.0001, ns P = 0.0738; ****P < 0.0001, ns P = 0.8588. (**e**) Quantification of cell proliferation of GCs from 3-NP-treated WT and *Ncoa7*^−*/*−^ mice treated with DMSO or rapamycin (n = 3 biological replicates per group). *P = 0.0351, *P = 0.0131. (**f**) Quantification of expression levels of SASP genes (*Il1b*, *Il8*, *Mmp3* and *Ccl2*) via RT‒qPCR in GCs isolated from 3-NP-treated WT and *Ncoa7*^−*/*−^ mice treated with DMSO or rapamycin (n = 4 biological replicates per group). ns P = 0.8979, ns P = 0.7816, ns P = 0.3738, ns P = 0.6572, *P = 0.0376, *P = 0.0321, **P = 0.0070, **P = 0.0045. (**g**) Quantification of the ovary index in 3-NP-treated WT and *Ncoa7*^−*/*−^ mice treated with DMSO or rapamycin (n = 8 biological replicates per group). ns P = 0.2740, ns P = 0.0695. Data in **c**-**g** are expressed as means ± s.e.m, and compared by two-tailed Student’s t-test. Source data

Rapamycin can stimulate autophagy and has been shown to prolong the lifespan of mice^40,41^. To test whether boosting autophagy could alleviate cellular senescence arising from SG accumulation, we examined the effects of rapamycin on granulophagy in *NCOA7*-KO and *NCOA7*-mutant knock-in KGN cells exposed to arsenate (Fig. 6a). Rapamycin treatment activated autophagic flux pathways and effectively abrogated cellular senescence in both *NCOA7*-deficient cells, indicated by decreased senescence markers and improved cellular growth kinetics (Fig. 6b–e and Extended Data Fig. 7a,b). Furthermore, granulophagy was indeed accelerated upon removal of stress conditions in rapamycin-treated *NCOA7*-KO cells (Extended Data Fig. 7c); however, rapamycin treatment failed to improve the SG clearance in *ATG7*-knockdown KGN cells (Extended Data Fig. 7d). Consistently, rapamycin treatment could also alleviate the senescent phenotype of ovarian GCs isolated from *Ncoa7*^−*/*−^ mice (Extended Data Fig. 7e,f).

To corroborate this for in vivo settings, we then examined whether rapamycin treatment could alleviate the ovarian aging induced in response to oxidative stress (Fig. 6a). In wild-type and *Ncoa7*^−*/*−^ mice pretreated with rapamycin or dimethylsulfoxide (DMSO) followed by 3 weeks of 3-NP administration, we found that *Ncoa7*^−*/*−^ mice treated with rapamycin had higher follicle counts, reduced ovarian fibrosis and decreased expression of senescence markers (SA-β-gal, p16^INK4a^ and p21^Cip1^) compared to the DMSO control group (Fig. 6f–i and Extended Data Fig. 7g). Collectively, these results suggest that modulating autophagic flux could potentially serve as an effective strategy to attenuate cell senescence and consequently alleviate ovarian aging caused by SG accumulation arising from NCOA7 deficiency or dysfunction.

### Targeted strategies alleviate GC senescence in ovarian aging

**Fig. 7:**
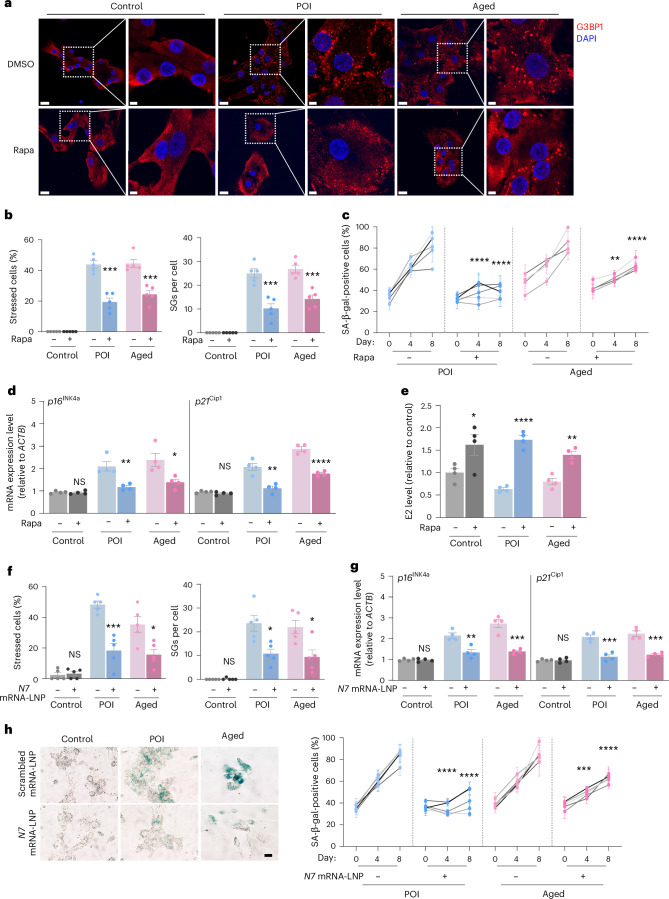
Clinical alleviation of ovarian aging by promoting autophagy or targeting NCOA7 to attenuate GC senescence. **a**,**b**, Immunofluorescence (**a**) and quantification (**b**) of SGs (*n* = 5 biological replicates per group) in GCs from control, POI and middle-aged participants treated with DMSO or rapamycin. Scale bars, 20 μm or 5 μm. *P* values (from left to right): ***P = 0.0001, ***P = 0.0005, ***P = 0.0007, ***P = 0.0006. **c**, Quantification of SA-β-gal-positive cells in GCs from POI (*n* = 5 biological replicates) and middle-aged (*n* = 4 biological replicates) participants treated with DMSO or rapamycin. ****P < 0.0001, ****P < 0.0001, **P = 0.0061, ****P < 0.0001. **d**, Expression levels of senescence markers (*p16*^*INK4a*^ and *p21*^*Cip1*^) in GCs from control, POI and middle-aged participants treated with DMSO or rapamycin (*n* = 4 biological replicates per group). NS, *P* = 0.8945; **P = 0.0059; *P = 0.0201; NS, *P* = 0.1368; **P = 0.0024; ****P < 0.0001. **e**, Analysis of E2 levels in the supernatant of GCs from control, POI and middle-aged participants treated with DMSO or rapamycin (*n* = 4 biological replicates per group). *P = 0.0494, ****P < 0.0001, **P = 0.0013. **f**, Quantification of SGs in GCs from control, POI and middle-aged participants treated with scrambled mRNA-LNPs or *N7* mRNA-LNPs (*n* = 5 biological replicates per group). NS, *P* = 0.6915; **P = 0.0004; *P = 0.0132; NS, *P* = 0.3466; *P = 0.0104; *P = 0.0143. **g**, Quantification of expression levels of senescence markers (*p16*^*INK4a*^ and *p21*^*Cip1*^) in GCs from control, POI and middle-aged participants treated with scrambled mRNA-LNPs or *N7* mRNA-LNPs (*n* = 4 biological replicates per group). NS, *P* = 0.7277; **P = 0.0047; ***P = 0.0006; NS, *P* = 0.9957; ***P = 0.0005; ***P = 0.0003. **h**, SA-β-gal staining and the percentage of SA-β-gal-positive cells in GCs from POI and middle-aged participants treated with scrambled mRNA-LNPs or *N7* mRNA-LNPs (*n* = 5 biological replicates per group). Scale bar, 20 μm. ****P < 0.0001, ****P < 0.0001, *P = 0.0150, ***P = 0.0002. Data in **b**–**h** are expressed as mean ± s.e.m. and compared by two-tailed Student’s *t*-test. Source data

**Extended Data Fig. 8:**
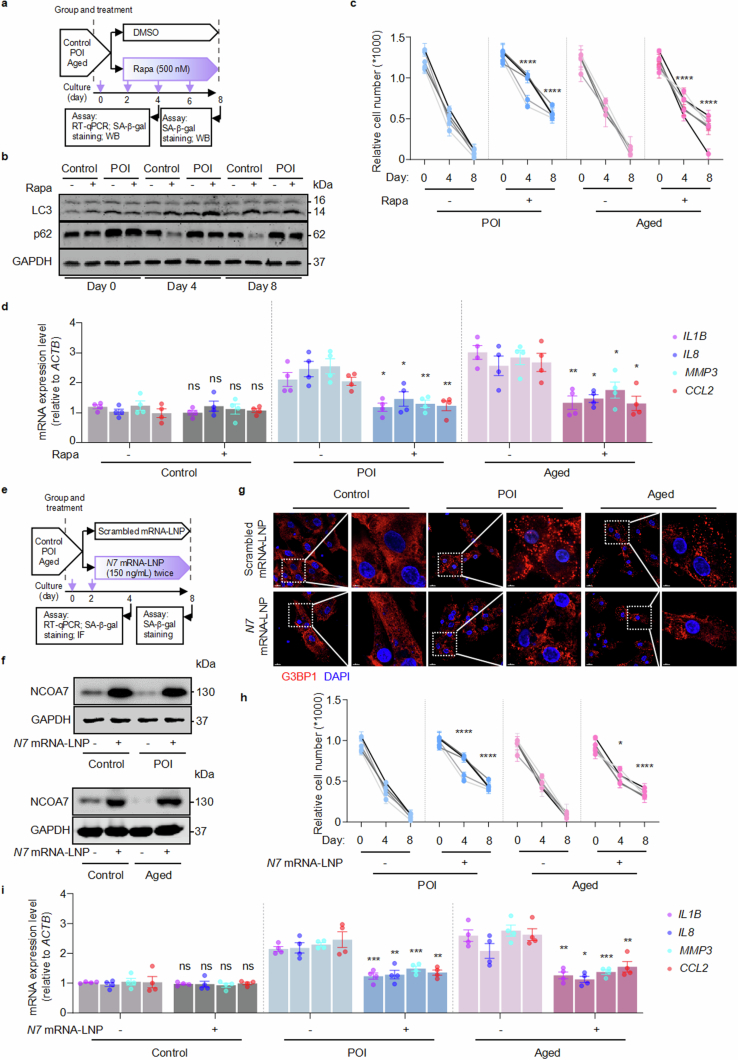
Promoting autophagy or targeting NCOA7 attenuates clinical GC senescence. (**a**) Schematic diagram showing the experimental design with rapamycin treatment in GCs from control, POI, and middle-aged participants. (**b**) Western blot of LC3 and p62 protein levels in GCs from control and POI women treated with DMSO or rapamycin. (**c**) Quantification of cell proliferation of GCs from POI and middle-aged participants treated with DMSO or rapamycin (n = 5 biological replicates per group). ****P < 0.0001, ****P < 0.0001, ****P < 0.0001, ****P < 0.0001. (**d**) Expression levels of SASP genes (*IL1B*, *IL8*, *MMP3* and *CCL2*) via RT‒qPCR in GCs from control, POI, and middle-aged participants treated with DMSO or rapamycin (n = 4 biological replicates per group). ns P = 0.0995, ns P = 0.3605, ns P = 0.6335, ns P = 0.5805, *P = 0.0140, *P = 0.0285, **P = 0.0042, **P = 0.0083, **P = 0.0020, *P = 0.0205, *P = 0.0238, *P = 0.0120. (**e**) Schematic diagram showing the experimental design for *N7* mRNA-LNP treatment in GCs from control, POI and middle-aged participants. (**f**) Western blot of NCOA7 protein levels in GCs from POI and middle-aged participants treated with scrambled mRNA-LNPs or *N7* mRNA-LNPs. (**g**) Immunofluorescence of SGs in GCs from control, POI, and middle-aged participants treated with scrambled mRNA-LNPs or *N7* mRNA-LNPs. Scale bar: 20 μm or 5 μm. (**h**) Quantification of cell proliferation of GCs from POI and middle-aged participants treated with scrambled mRNA-LNPs or *N7* mRNA-LNPs (n = 5 biological replicates per group). ****P < 0.0001, ****P < 0.0001, *P = 0.0133, ****P < 0.0001. (**i**) Expression levels of SASP genes (*IL1B*, *IL8*, *MMP8*, *CCL2*) in GCs from control, POI and middle-aged participants treated with scrambled mRNA-LNPs or *N7* mRNA-LNPs (n = 4 biological replicates per group). ns P = 0.1461, ns P = 0.9336, ns P = 0.3977, ns P = 0.8452, ***P = 0.0005, **P = 0.0069, ***P = 0.0003, **P = 0.0069, **P = 0.0010, *P = 0.0120, ***P = 0.0005, **P = 0.0067. Data in **c**-**d** and **h**-**i** are expressed as means ± s.e.m, and compared by two-tailed Student’s t-test. Source data

Given the above data, we next tested whether boosting autophagy could also reduce ovarian GC senescence in participants with POI and middle-aged participants. To this end, we treated GCs isolated from participants with POI, middle-aged and control participants with rapamycin or DMSO (Extended Data Fig. 8a). Rapamycin treatment resulted in notably enhanced autophagic flux on days 4 and 8, and decreased SG accumulation in GCs from individuals with POI and middle-aged participants (Fig. 7a,b and Extended Data Fig. 8b). Consistently, rapamycin-treated GCs exhibited alleviated senescent phenotypes, including reduced proportions in SA-β-gal-positive cells and higher proliferation rates on day 4 and day 8, as well as decreased *p21*^*Cip1*^, *p16*^*INK4a*^ and SASP marker (*IL1B*, *IL8*, *MMP3* and *CCL2*) expression compared to the DMSO controls (Fig. 7c,d and Extended Data Fig. 8c,d). Notably, E2 production was also significantly higher following rapamycin treatments (Fig. 7e). These results suggest that rapamycin treatment could accelerate the elimination of SGs to alleviate GC senescence and ultimately ovarian aging in humans.

Further, we asked whether direct regulation of NCOA7 expression could more generally attenuate the cellular senescence phenotype of individuals with POI and middle-aged participants. Lipid nanoparticle (LNP)-mRNA formulations produced by good manufacturing practices have been reported to exhibit reasonable stability, safety and shelf-life, and could be successfully applied in clinics to deliver targeted mRNA therapies^42^. We thus prepared a formulation of mRNAs encoding human *NCOA7* in an LNP vehicle (*N7* mRNA-LNP) for therapeutic investigation. The ovarian GCs were isolated from individuals with POI, middle-aged and control participants, and cultured with *N7* mRNA-LNP or scrambled mRNA-LNP (Extended Data Fig. 8e). Western blotting showed that *N7* mRNA-LNP effectively increased the expression of NCOA7 in all groups (Extended Data Fig. 8f). Consistently, granulophagy was accelerated in GCs isolated from individuals with POI and middle-aged participants who were treated with *N7* mRNA-LNP (Fig. 7f and Extended Data Fig. 8g). Furthermore, GCs from individuals with POI and middle-aged participant displayed improved senescence phenotypes upon delivery of *N7* mRNA-LNP relative to the control group, as evidenced by increased proliferation and diminished proportions of SA-β-gal-positive cells as well as decreased *p16*^*INK4a*^, *p21*^*Cip1*^ and SASP marker expression (Fig. 7g,h and Extended Data Fig. 8h–i). Therefore, these data indicate that LNP-mRNA delivery to elevate NCOA7 expression can effectively accelerate granulophagy and attenuate GC senescence, supporting promising potential of this therapeutic strategy to alleviate ovarian aging.

## Discussion

Oxidative stress has been identified as a source of vicious circle feedback that contributes to ovarian dysfunction and diminished ovarian reserve^11^; however, the mechanism linking chronic oxidative stress to ovarian aging has remained unknown. Here, we found deleterious mutations and downregulated expression in the oxidative stress-response gene, *NCOA7*, in women with POI or physiological ovarian aging that recapitulate the senescent phenotype of GCs, thus providing the first evidence linking NCOA7 with ovarian aging. NCOA7 ablation or silencing in KGN cells or mice results in a senescent phenotype that resembles ovarian aging in humans, characterized by declined fecundity, attenuated E2 production, increased follicle exhaustion and ovarian fibrosis, which can be further exacerbated upon oxidative stress. This cumulative evidence supports that NCOA7 deficiency promotes senescence in ovarian GCs under oxidative stress, accelerating ovarian aging in both humans and mice.

NCOA7 has been previously reported to interact with V-ATPase to mitigate the effects of oxidative stress^43^. In this study, we mapped the whole NCOA7 interactome, which revealed an NCOA7–G3BP1–V-ATPase interaction complex that could explain the key function of NCOA7 in mitigating ovarian aging. Defects in SG metabolism have been identified in aging and aging-associated disorders, such as neurodegenerative diseases^12,18,44,45^. We observed that SG accumulation is aberrantly increased in GCs from both participants with POI and middle-aged research participants, as well as global and GC-specific *Ncoa7*-KO mice. Furthermore, we found that NCOA7 deficiency specifically impairs SG clearance, but not formation. Our data thus depict a mechanism of dysregulated SG metabolism mediated by NCOA7 deficiency that underlies ovarian aging, consequently expanding our understanding of how ovarian resilience to different stress stimuli helps maintain reproductive competence and longevity. Further studies exploring the role of NCOA7 in GCs modulating folliculogenesis and ovarian microenvironment are warranted.

Although SG dynamics have been extensively studied, the mechanisms governing their disassembly and clearance have remained unclear. In particular, whether autophagy mediates clearance of excess SGs remains controversial^17,46,47^. We identified NCOA7 as an adaptor for SG autophagic clearance. On the one hand, NCOA7 can directly interact with and promote the LLPS of G3BP1 to permit substrate binding with SGs. On the other hand, NCOA7 associates with LC3B within autophagosomes, allowing substrate binding to autophagic machinery. Although the direct immunofluorescent colocalization of LC3 with SGs is lacking, we could clearly show SGs localized within lysosomes, which serve as the primary degradative compartment in the autophagy pathway. The lysosomal inhibitor chloroquine mainly inhibits autophagy by impairing autophagosome fusion with lysosomes, resulting in SG accumulation. Conversely, rapamycin activates autophagic flux, which could promote autophagic clearance of SGs and alleviate the senescent phenotype in *NCOA7*-KO cells and mice. These findings suggest that NCOA7 may play an essential role in restoring cellular homeostasis following stress via autophagy-dependent clearance of SGs in GCs, which is pivotal for ovarian function and fertility competence.

In light of this abovementioned mechanism, it is likely that either granulophagy or NCOA7 per se may serve as potentially effective therapeutic targets for future strategies that profoundly improve female health and longevity. Rapamycin has been shown to confer several benefits in ameliorating aging, extending both reproductive and overall lifespan in female rodents^48–50^. Our results indicate that boosting autophagy by rapamycin treatment can indeed inhibit GC senescence and alleviate ovarian aging, apparently by promoting SG clearance, in both mouse and human GCs; therefore, further exploration of this strategy is warranted to potentially improve therapeutic outcomes for patients with pathological or physiological ovarian aging. Of note, rapamycin can also generally induce macroautophagy, resulting in enhanced clearance of numerous cytoplasmic substrates including SGs. Besides, our findings demonstrate that a targeted increase in ovarian NCOA7 expression, such as through delivery of *N7* mRNA-LNP, can also stimulate SG clearance to de-escalate oxidative stress; however, more extensive, preclinical studies examining the efficacy of this personalized medicine strategy in vivo, and the mechanisms through which it affects oxidative stress response, are necessary. Validation of such a nanotherapeutic intervention may hold considerable promise for treating female-factor subfertility and diminished ovarian reserve, or preserving fertility in individuals at high risk of premature menopause.

Despite these interesting findings, some unaddressed questions remain. For instance, although NCOA7 can interact with V-ATPase in its protective mechanism against cellular senescence, it remains unknown whether V-ATPase also directly participates in granulophagy. In addition to the cytoplasmic functions of NCOA7 examined in our current work, whether and how it exhibits nuclear functions that contribute to its anti-aging effects have not been documented. The differences in NCOA7 localization have been observed across different cell types^51,52^, such localization disparities may lead to the non-universality of the regulatory mechanisms governing SGs mediated by NCOA7 across diverse cellular contexts. It should be noted that NCOA7-related mechanisms cannot explain all cases of ovarian aging in clinic. In fact, a subset of individuals with ovarian aging shows no changes in NCOA7 expression, implying a more complex pathological mechanism. Regarding the therapeutic options, numerous studies have shown that rapamycin can extend lifespan across various organisms^48–50^, the most well-understood mechanism entails suppression of mTORC1 signaling; however, it is likely that rapamycin may exert more complicated interactions in vivo, and closer scrutiny of its mode of action is certainly warranted.

In summary, this study defines NCOA7 as an adaptor protein required for autophagic clearance of SGs in response to oxidative stress stimuli, thereby protecting cells from premature senescence and alleviating ovarian aging. These findings expand our understanding of the cellular molecular events leading to premature or physiological ovarian aging, and suggest that therapeutic approaches targeting NCOA7 and/or SG turnover could potentially mitigate ovarian aging and extend reproductive longevity.

## Methods

### Human participants and samples

All participants were recruited from the Hospital for Reproductive Medicine Affiliated with Shandong University (SDU) from July 2021 to July 2024. All procedures involving human participants in this study were approved by the Institutional Review Board of Center for Reproductive Medicine, SDU (2020IRB18 and 2023IRB26). Written informed consent was provided by each participant. The inclusion criteria for POI included secondary amenorrhea for at least 4 months and a serum basal FSH concentration >25 IU l^−1^ (on two occasions >1 month apart) before the age of 40 years, in accordance with the European Society of Human Reproduction and Embryology (ESHRE) and Chinese guidelines^53,54^. Preclinical POI was defined as regular or irregular menses, increased basal serum FSH (10 IU l^−1^ < FSH ≤ 25 IU l^−1^, on two occasions >4 weeks apart) and an antral follicle count (AFC) < 5 before the age of 40 years, as previously reported^55,56^. The advanced-aged premenopausal (middle-aged) group included women older than 40 years before menopause. Patients with regular menstrual cycles and normal FSH levels (<10 IU l^−1^) who were seeking infertility treatment due to tubal obstruction or male factors were recruited as controls. Individuals with chromosomal abnormalities, known gene mutations, a history of ovarian surgery, radio- or chemotherapy, a history of recurrent spontaneous abortion, endometriosis or autoimmune disease, or infection in the previous 3 months were excluded. The clinical characteristics of the participants are summarized in Supplementary Table 2.

The control and middle-aged ovarian samples were obtained with bilateral salpingo-oophorectomy due to endometrial cancer and those of individuals with POI were collected from donors with ovarian biopsy. Fetal samples of the brain, heart, liver, spleen, lung, kidney and ovary were obtained from human fetuses (abortion induced at 22 weeks). Human GCs were isolated and purified from donors with preclinical POI, middle-aged individuals and controls undergoing in vitro fertilization (IVF) or intracytoplasmic sperm injection and embryo transfer (ICSI-ET). On the day of oocyte retrieval, after the cumulus cells tightly surrounding the oocytes were transferred together with the oocytes for subsequent fertilization or preservation, the follicular fluid of large follicles (>14 mm) containing mural GCs was collected, and the precipitates were collected after centrifugation followed by incubation with hyaluronidase (80 IU ml^−1^; Solarbio) for 30 min at 37 °C. The components were then transferred to lymphocyte separation medium (MP Biomedicals) for horizontal centrifugation at 330*g* for 10 min. Once isolated from the interlayer phase, the GCs were washed in phosphate-buffered saline (PBS) for corresponding experiments (such as RNA-seq and RT–qPCR). If needed, the isolated GCs were seeded in dishes for further culture in DMEM/F12 (Gibco) supplemented with 5% FBS (Gibco) and 1% penicillin‒streptomycin (Gibco) at 37 °C in a humidified atmosphere of 5% CO_2_. The purity and viability of the isolated GCs were assessed via immunofluorescence staining for GC markers (aromatase and FSHR) and flow cytometry using viability dyes.

### Mice and mouse samples

Wild-type C57BL/6J mice were routinely used if not specifically indicated. C57BL/6J-*Ncoa7*^−*/*−^ mice, generated via CRISPR–Cas9 technology, were obtained from Cyagen Bioscience. The *Ncoa7*^*flox/flox*^ mice were purchased from Gempharmatech Co. The *Foxl2*^*cre*^ knock-in mice were obtained from F. Gao, Institute of Zoology, Chinese Academy of Sciences. The *Foxl2*^*cre*^ mice were crossed with *Ncoa7*^*flox/flox*^ mice to generate *Foxl2*^*cre*^; *Ncoa7*^*flox/flox*^ mice. Genotyping was assessed by PCR using mouse tail DNA (for primer sequences, see Supplementary Table 4). Six-week-old wild-type and *Ncoa7*^−*/*−^ female mice were administered 3-NP (40 mg kg^−1^ day^−1^, diluted with normal saline, Sigma) every day at 12:00 for 21 consecutive days. For rapamycin therapy (5 mg kg^−1 ^day^−1^), 28 consecutive days of intraperitoneal injections were initiated 1 week before 3-NP treatment. All mice were housed on a 12-h light–dark cycle at 25 °C and were provided with standard chow and water ad libitum. The animal protocols and experiments were approved by the animal protocol guidelines of SDU and approved by the Institutional Review Board of the Center for Reproductive Medicine, SDU (2020IRB18 and 2023IRB26).

Ovary tissues were collected from 8-week-old and 10-month-old female mice. For ovarian GC isolation, female mice at 3 weeks old were intraperitoneally injected with 5 IU of pregnant mare serum gonadotropin (Ningbo Sansheng) to stimulate follicle growth. After 24 h, the mice were killed and the ovaries were rapidly isolated into a dish with medium under aseptic conditions. To release the GCs, the ovaries were carefully pricked with an insulin syringe needle. The GCs were washed by centrifugation at 200*g* for 5 min and then resuspended for corresponding experiments. If needed, the isolated GCs were cultured in DMEM/F12 (Gibco) supplemented with 5% FBS (Gibco) and 1% penicillin‒streptomycin (Gibco) in a humidified atmosphere of 5% CO_2_ at 37 °C for 12 h to be adherent for subsequent treatment. The purity and viability of the isolated GCs were assessed via immunofluorescence staining for GC markers and flow cytometry using viability dyes.

### Cell lines

Experiments were performed using KGN (RIKEN BioResource Center, RCB1154), HEK293T (National BioMedical Cell-line Resource, 3101HUMSCSP502) and HeLa (Cell Resource Center, Institute of Basic Medical Sciences, CAMS/PUMC) cell lines. Cells were cultured in DMEM/F12 (Gibco) or high-glucose DMEM (BI) supplemented with 10% FBS (Gibco) and 1% penicillin‒streptomycin (Gibco) and maintained in a humidified 5% CO_2_ incubator at 37 °C unless otherwise indicated. All cell cultures tested negative for *Mycoplasma* contamination.

*NCOA7*-KO KGN cells were generated via the CRISPR–Cas9 system. In brief, all guide RNAs were designed in Benchling using the CRISPR Design Tool and subsequently cloned and inserted into lentiCRISPR-v2. To produce lentivirus in HEK293T cells, the DNA mixture consisted of 2.5 μg of the lentiCRISPR-v2 plasmid, 2 μg of the psPAX2 plasmid and 1.5 μg of the pMD2.G plasmid was transfected with jetOPTIMUS DNA Transfection Reagent (Polyplus). After 10 h, the medium was replaced with fresh DMEM. After 24 h, the lentivirus-containing supernatant was collected, filtered and stored at −80 °C. For lentiviral transfection, 1 × 10^5^ KGN cells were seeded into a 12-well plate (Costar) and lentivirus and 10 µg ml^−1^ Polybrene (Beyotime) were added. After 24 h of infection, the cells were selected with 1 µg ml^−1^ puromycin for 48 h. The monoclonal cell population was isolated by infinite dilution. The KO efficiency was tested at the protein level via western blotting. If not indicated otherwise, *NCOA7*-KO was performed via KO1 cells. The sequences of the sgRNAs used to target *NCOA7* are shown in Supplementary Table 4.

### Gene variation filtration

The in-house WES database of individuals with POI was established as previously reported^27^. The variations in the exonic or splice region of the *NCOA7* gene were screened, and common variants with minor allele frequency > 0.001 either in public controls from the 1000 Genomes Project (https://www.internationalgenome.org/), ExAC (https://ngdc.cncb.ac.cn/databasecommons/database/id/3774) or gnomAD (https://gnomad.broadinstitute.org/) were filtered out. Variations that altered protein sequences (nonsense mutations, missense mutations, canonical splicing site variants and coding indels) and were predicted to be potentially deleterious by more than four software programs (SIFT, PolyPhen-2, MutationTaster, CADD, DANN and MetaSVM) were retained for further analysis. The variants were classified as pathogenic, likely pathogenic or variants of uncertain significance according to the guidelines proposed by the American College of Medical Genetics and Genomics/Association for Molecular Pathology^57^. The candidate variants were confirmed by Sanger sequencing.

### Minigene assay

The *NCOA7* c.699+3A>G variant is located at the donor splice site of intron 6. The intron 6 (315 bp)-exon 7 (126 bp)-intron 7 (483 bp) fragments were acquired via PCR and integrated into the pcMINI vector. HeLa and HEK293T cells were transfected with the wild-type or c.699+3A>G vector, respectively. After culturing for 48 h, total RNA was extracted via TRIzol (TaKaRa) and complementary DNA was obtained via HifairTM first Strand cDNA Synthesis SuperMix (TEASEN). The primers used for constructing the minigene vectors and for detecting alternative splice sites are listed in Supplementary Table 4.

### Plasmid cloning

The plasmids, pcDNA3.1-3×Flag-NCOA7, pcDNA3.1-HA-ATP6V1A and pcDNA3.1-HA-ATP6V1B2, were purchased from UniBio Biotechnology Co. All the remaining plasmids used were prepared as described previously, unless stated otherwise^58^.

For pCDH-copGFP-NCOA7, *NCOA7* was amplified via PCR from cDNA from the human GCs as a template and inserted into the pCDH-CMV-MCS-EF1-CopGFP vector via homologous recombination via NheI. The NCOA7^c.G111A^ construct was generated with a site-directed mutagenesis kit, with either the pCDH-CMV-MCS-EF1-CopGFP-NCOA7 or the pcDNA3.1-3×Flag-NCOA7 plasmid serving as a template. The plasmid was subsequently used as a template to block the CRISPR/Cas9 mutation of NCOA7. Four variants of *NCOA7* (NM_001199620: c.67C>T, p.Q23X; c.382G>A, p.A128T; c.437C>T, p.T146I; c.2379G>T, p.W804C) were generated by site-directed mutagenesis with a Mut Express II Fast Mutagenesis kit (Vazyme), using the pcDNA3-TO-N′Flag-NCOA7^c.G111A^ plasmid as a template. The resulting plasmids were pcDNA3.1-3×Flag-NCOA7^p.Q23X^, pcDNA3.1-3×Flag-NCOA7^p.A128T^, pcDNA3.1-3×Flag-NCOA7^p.T146I^ and pcDNA3.1-3×Flag-NCOA7^p.W804C^. The NCOA7 c.699+3A>G variant (pcDNA3.1-CopGFP-NCOA7^p.Gly217Glyfs*12^) plasmid was obtained from UniBio.

The Flag-NCOA7-ΔTLDc (lacking amino acids 780–942) and Flag-NCOA7-ΔLysM (lacking amino acids 122–158) constructs were generated via site-directed mutagenesis by ApexHF HS DNA polymerase (Accurate Biology) using pcDNA3.1-3×Flag-NCOA7 as templates. The pCDH-NCOA7^c.G111A^-ΔLysM (lacking amino acids 122–158) construct was generated via PCR with ApexHF HS DNA polymerase (Accurate Biology) and pCDH-CMV-MCS-EF1-CopGFP-NCOA7^c.G111A^ was used as a template.

G3BP1, LARP1, CAPRIN1, ATP6V1A, ATP6V1B2 and ATP6V1D were amplified via PCR using cDNA from KGN cells as a template and inserted into the pcDNA3-TO-N′HA vector (HA–empty) or pcDNA3-TO-N′myc vector (myc–empty) via restriction-ligation cloning. The HA-G3BP1-ΔNTF2L (lacking amino acids 1–139) construct was generated via PCR using ApexHF HS DNA polymerase (Accurate Biology) and the vector pcDNA3-TO-N′HA containing G3BP1 (HA–G3BP1) was used as a template.

### Generation of *NCOA7* heterozygous variants in *NCOA7*-KO KGN cells

Three *NCOA7* missense mutant plasmids (pcDNA3.1-3×Flag-NCOA7^p.A128T^, pcDNA3.1-3×Flag-NCOA7^p.T146I^ and pcDNA3.1-3×Flag-NCOA7^p.W804C^) were generated as mentioned above. *NCOA7*-KO KGN cells were transfected with 2 μg each of wild-type or mutant *NCOA7* plasmids at a 1:1 ratio to mimic the human heterozygous status. After transfection for 24 h, the medium was replaced with fresh medium and cell experiments were performed on days 0, 4 and 8. Day 0 was defined as 48 h after plasmid transfection. The cell senescence phenotypes were shown at 4 days after transfection, unless otherwise noted.

### *NCOA7* re-expression in *NCOA7*-KO cells

*NCOA7*-KO KGN cells (2 × 10^5^) were seeded in six-well plates for 24 h before transfection. The pCDH-copGFP-NCOA7^c.G111A^, pCDH-copGFP-NCOA7^c.G111A^-ΔLysM and vector plasmids were constructed as described above and subsequently subjected to a lentivirus packaging assay. For infection, NCOA7 lentivirus was added to the cell medium for 12 h and then replaced with fresh culture medium. Stable NCOA7-overexpressing and NCOA7 domain-truncated cells were sorted using fluorescence-activated cell sorting.

### Cycloheximide chase assay

A cycloheximide (CHX) chase assay was conducted to evaluate the protein stability of NCOA7 wild-type and mutant constructs (p.A128T, p.T146I and p.W804C) in HEK293T cells. HEK293T cells were plated in a six-well plate and transfected with NCOA7–Flag wild-type or mutant constructs for 48 h. Then the cells were treated with CHX at a concentration of 100 μg ml^−1^ and collected for western blot at 0, 1.5, 3, 6, 12 and 24 h to evaluate changes in NCOA7 protein expression.

### RNA-seq and data analysis

Freshly isolated human ovarian GCs from patients with POI and control participants were analyzed via RNA-seq. Total RNA was extracted by using TRIzol reagent (Thermo Scientific) and whole-genome gene expression analysis was performed. mRNA was purified from total RNA by using poly-T oligo-attached magnetic beads and cDNA was synthesized. To preferentially select cDNA fragments 370–420 bp in length, the library fragments were purified with the AMPure XP system (Beckman Coulter). After PCR amplification, the PCR products were purified with AMPure XP beads, after which the library was finally obtained. The different libraries were pooled according to the effective concentration and the target amount of data collected off the machine and subsequently sequenced on the Illumina NovaSeq 6000 platform. All DEGs were subjected to KEGG enrichment analysis and a volcano plot was generated. DEGs are listed in Supplementary Table 6.

### RT‒qPCR

Cells and tissues from humans or mice were lysed in TRIzol reagent (Sigma) and total RNA was extracted via a Rapid RNA Extraction kit (Accurate Biology) following the manufacturer’s protocol. The integrity of the RNA was verified via agarose gel electrophoresis and quantified via a NanoDrop spectrophotometer. The cDNA was synthesized via an Evo M-MLV RT kit (Accurate Biology) with 1 µg of template RNA per reaction. RT‒qPCR was performed in an Eppendorf Mastercycler ep realplex with the appropriate primers at a final concentration of 500 nM and a volume of cDNA corresponding to 5 ng of RNA per reaction. The relative expression levels were calculated via the 2−ΔΔCt method. Primer sequences are listed in Supplementary Table 4.

### Western blotting

The cells were washed with PBS and collected with RIPA lysis buffer (Beyotime) containing phenylmethyl sulfonyl fluoride and a protease cocktail. Mouse ovary protein was extracted with a Minute Total Protein Extraction kit (Invent). The protein concentration was measured via a BCA protein assay kit (Thermo Scientific). Equal volumes (50 μg) of protein lysates were mixed with 1× loading buffer (Beyotime), heated for 5 min at 100 °C, separated by SDS‒PAGE and subsequently transferred onto nitrocellulose filter membranes or PVDF membranes. After being blocked in 5% (w/v) skim milk for 1 h at room temperature, the membranes were incubated overnight with primary antibodies at 4 °C. The next day, the membranes were incubated for 1 h with the corresponding HRP-conjugated secondary antibodies, after which the proteins were detected with a ChemiDoc MP Imaging System (Bio-Rad). Protein expression was quantified via ImageJ v.1.8.0. The antibodies used are listed in Supplementary Table 5.

### Cell counting and cell proliferation assay

For the cell counting assay, 5 × 10^3^ KGN cells or 1 × 10^3^ GCs were seeded in 96-well plates on day 0 and digested with trypsin (Genview) on days 4 and 8. For KGN cell counting, the resulting cell suspension was subsequently diluted 1:1 with 0.2% trypan blue and a 20-µl mixture was injected into a Countstar chamber slide and analyzed with a Countstar. In GCs counting experiment, cells were digested with trypsin on days 4 and 8, and the resulting suspension was transferred to a hemocytometer for counting under a microscope.

For the CCK8 assay, 1 × 10^3^ GCs per well were seeded into a 96-well plate and incubated with CCK8 (Beyotime, 10 μl per well) for 2 h at 37 °C on days 0, 1, 2 and 4. The absorbance was subsequently measured at 450 nm with a microplate reader (BioTek, Synergy).

### SA-β-gal measurement

SA-β-gal staining was performed following the manufacturer’s protocol (Solarbio). The cells or mouse ovarian tissue sections were washed twice with PBS and fixed for 15 min at room temperature. Samples were then incubated with β-galactosidase staining solution at 37 °C overnight. An optical microscope (Olympus) was used to observe SA-β-gal-stained cells, and the percentage of SA-β-gal-positive cells was calculated via ImageJ v.1.8.0. At least three biological replicates were performed for each group and more than 100 cells were quantified in each replicate.

For the fluorescence assay, SA-β-gal was stained by using a SPiDER-βGal probe (DOJINDO) in accordance with the manufacturer’s protocol, followed by immunofluorescence staining of p21^Cip1^. The images were acquired with a confocal laser-scanning microscope (Andor Dragonfly 200).

To quantify the activity of SA-β-gal, a quantitative chemiluminescence assay was conducted. In brief, cells were collected, lysed and incubated with a galactosidase substrate for 40 min at room temperature. After adding 300 μl of Emerald luminescence amplifier, the samples were measured using a luminometer. Protein quantification was achieved with Bradford assays.

### Three-dimensional structural analysis

The three-dimensional structures of wild-type NCOA7 were constructed by AlphaFold 2.3 and V-ATPase and G3BP1 were extracted from the RCSB Protein Data Bank (P38606 and Q13283). The structures of five NCOA7 mutant (p.Q23X, p.A128T, p.T146I, p.W804C and p.Gly217Glyfs*12) proteins were constructed via the mutagenesis module in PyMOL. Protein‒protein docking was performed with Rosetta software to explore the possible binding interface between the wild-type NCOA7, V-ATPase and G3BP1 proteins. A maximum of 100 docking conformations was considered, and the best conformation with the lowest binding energy was selected. Position-restrained molecular dynamics simulations were carried out with AMBER16 software to eliminate steric conflicts within each protein system. One 100 ns molecular dynamics simulation was performed for protein‒protein complexes and the final average structures were extracted for binding mode and affinity analyses.

### Immunofluorescence

Cells were grown on 35-mm confocal small dishes (NEST) before staining. After washing with PBS three times, cells were fixed with 4% paraformaldehyde (Solarbio), permeabilized with 0.5% Triton X-100 (Solarbio) for 10 min, blocked with 5% BSA (Solarbio) for 1 h at room temperature and incubated with primary antibodies at 4 °C overnight. Cells were then washed with PBST and incubated with fluorescent secondary antibodies for 1 h at room temperature. Finally, cells were stained with 4′,6-diamidino-2-phenylindole (DAPI) (250 ng ml^−1^, Thermo Scientific) before imaging. All fluorescence images were acquired with a confocal laser-scanning microscope (Andor Dragonfly 200) and quantitative analysis of fluorescence intensity was performed with Imaris Viewer v.10.2.0 and ImageJ v.1.8.0, measuring the average intensity of each fluorescent channel within regions of interest in the chosen fields after background subtraction. At least three independent experiments were conducted. The antibodies used are listed in Supplementary Table 5.

### ELISA

The concentrations of IL-1β, IL-8, MMP3 and CCL2 from wild-type, *NCOA7*-KO and *NCOA7*-rescued KGN cell supernatants were assayed by using the human IL-1 β/IL-1F2 Quantikine ELISA kit (R&D Systems, DLB50), the human IL-8/CXCL8 Quantikine ELISA kit (R&D Systems, D8000C), the human MMP3 Quantikine ELISA kit (R&D Systems, DMP300) and the human CCL2/MCP-1 Quantikine ELISA kit (R&D Systems, DCP00), according to the manufacturers’ protocols. In brief, wild-type, *NCOA7*-KO and *NCOA7*-rescued cells were seeded in six-well plates and the supernatants were collected and probed for the levels of MMP3, IL-8, CCL2 and IL-1β proteins via ELISA. A microplate reader (BioTek) was used to assess the optical density value at 450 nm.

### Hormone measurement

To measure the E2 level in the cell culture supernatants, cells were seeded into six-well plates and cultured with fresh medium (10% charcoal-stripped FBS and 90% DMEM/F12 medium without phenol red). After incubation with 10 nM testosterone (Sigma) for 36 h, the supernatant was collected and diluted with DMEM/F12 without phenol red (Gibco) to a final volume of 500 µl and analyzed via the electrochemiluminescence method on the Cobas e601 analyzer (Roche Diagnostics).

To measure the serum hormone levels in the mice, the mice at the diestrus stage were killed and blood was collected. Serum was isolated by centrifuging at 600*g* at 4 °C for 15 min after incubation overnight. FSH and E2 concentrations were measured via radioimmunoassay (Beijing North Institute of Biotechnology).

### ROS detection assay

Cells were stained with 10 nM DCFH-DA (Beyotime) for 30 min at 37 °C according to the manufacturer’s instructions and then visualized via a confocal laser-scanning microscope (Andor Dragonfly 200).

### *NCOA7*-SunTag cell line and luciferase assay

To generate the *NCOA7*-SunTag reporter, the cDNA of human *NCOA7* 5′UTR was inserted into the SunTag-Renilla-MS2 plasmid (Addgene plasmid 119945) using HindIII and RsrII restriction sites. The *NCOA7*-SunTag reporter was stably integrated into KGN cells, which stably expressed scFv-GFP against GCN4 and the NLS-stdMCP-stdHalo fusion protein. NCOA7-SunTag-labeled KGN cells were generated and maintained as described below. In brief, genomic integration was achieved via recombinase-mediated cassette exchange (RMCE). A total of 3 × 10^5^ KGN cells were seeded into a six-well plate and were transfected the next day with 2 μg of the FLPe recombinase plasmid (Addgene) and 2 μg of the plasmid that carries the Flp recombinase target sites flanking the SunTag reporter. Puromycin (1 μg ml^−1^) was added to select the transfected cells. To obtain cells with successful RMCE, the medium was removed and replaced with 50 μM ganciclovir-containing medium for 10 days. Single cells were sorted into a 96-well plate, and a single-clone cell line was generated. The cells were subsequently expanded, and reporter expression was tested via a Renilla Luciferase Reporter Gene Assay kit (Beyotime). KGN cells harboring *NCOA7*-SunTag were seeded in 24-well plates and incubated with 1 μg ml^−1^ doxycycline for 2 h, followed by arsenate treatment for 1 h. The cells were subsequently lysed with 150 μl of lysis buffer per well. Then, 30 μl of lysate were transferred to a 96-well plate. Bioluminescence measurements were performed after the addition of Renilla luciferase assay reagent to each well.

### CUT&Tag library generation and high-throughput sequencing

The CUT&Tag assay was performed with a Hyperactive Universal CUT&Tag Assay kit for Illumina (Vazyme Biotech, China; TD903). In brief, GCs from periclinal POI or control participants were washed with washing buffer and then incubated with 10 μl of prewashed ConA beads in a 0.2-ml low-binding eight-strip tube. Then, 50 μl of antibody buffer supplemented with 1 μg of H3K27ac antibody were added and the samples were incubated overnight at 4 °C. The no-antibody control was incubated without primary antibody. The samples were incubated in 50 µl of DIG wash buffer supplemented with 0.6 μg of secondary antibody for 1 h at room temperature. After washing three times with DIG wash buffer, 100 µl of DIG-300 buffer with 2 μl of pA/G-Tnp was added and the samples were incubated at room temperature for 1 h. The samples were then washed three times with DIG-300 buffer. Then, 50 μl of tagmentation buffer were added to the samples, which were subsequently incubated at 37 °C for 1 h. The reaction was stopped with 5 μl of proteinase K, 100 μl of buffer L/B and 20 μl of DNA extraction beads. The samples were washed once with WA buffer and twice with WB buffer. DNA was extracted in ultrapure water and PCR was performed to amplify the libraries. All the libraries were sequenced on an Illumina NovaSeq platform according to the manufacturer’s instructions. All CUT&Tag data were mapped to the mm10 genome (GCF_000001635.26) via BWA v.0.7.12. All low-quality reads and PCR adaptors were removed. The BamCoverage command from deepTools v.3.0.2 was used to generate the track files. All peak calling was performed with MACS2 v.2.1.2 via the options ‘macs2 -q 0.05 –call-summits –nomodel –shift −100 –extsize 200 –keep-dup all’. The genomic distribution of CUT&Tag peaks was annotated with the R package ChIPseeker. Heatmaps and metaplots were generated for the protein-coding genes or specific peaks via deepTools (v.3.0.2) (ref. ^59^).

### Apoptosis assay

Cell apoptosis was assessed using annexin V-APC/7-AAD staining assays. In brief, cells were collected and washed with PBS. Apoptosis staining was performed using an annexin V-APC/7-AAD Apoptosis Detection kit (Liankebio) according to the manufacturer’s instructions. The stained cells were analyzed using a BD LSRFortessa, FACSDiva v.8.0 and FlowJo v.10.5.3.

### Fertility testing

Wild-type and *Ncoa7*^−*/*−^ or flox/flox and *Ncoa7*-cKO female mice (8 weeks old) were mated with fertile wild-type C57BL/6J male mice (8 weeks old) for 8 months and the number and timing of the litters were recorded.

### Ovarian index, ovarian histology and follicle counting

The mice were weighed and killed and the main organs in the hypogastrium were exposed via a U-shaped incision. The ovaries were isolated, placed on dry sterile gauze and weighed on an electronic analytical balance when no liquid remained. The ovarian index was calculated as the ovarian wet weight (mg)/body weight (g) × 100%. Bouin-fixed, paraffin-embedded ovarian tissue was serially sectioned at a thickness of 5 μm. After hematoxylin and eosin staining, every fifth section was analyzed in a double-blind manner by two researchers via an unbiased stereological method to determine the presence of oocytes and follicles. Primordial, primary, secondary and antral follicles were classified according to the corresponding histological morphology and only follicles containing a visible nucleus were counted. Five times the count value was taken as the result.

### Immunohistochemistry and PRS staining

For immunohistochemistry, mouse ovary sections were stained with anti-p16^INK4a^ or anti-p21^Cip1^ (1:100 dilution). For collagen staining, tissue sections were stained with Sirius red with a Picro Sirius Red Stain kit (Maokangbio). Images were taken under a microscope (OLYMPUS) and analyzed with ImageJ v.1.8.0.

### Analysis of the NCOA7 interactome

HEK293T cells were seeded in 10-cm dishes for 24 h and transfected with 10 µg of NCOA7-3×Flag plasmid for 48 h via polyplus transfection before pulldown experiments. The cells were washed with PBS and collected with IP lysis buffer (Beyotime) containing phenylmethyl sulfonyl fluoride and a protease inhibitor cocktail. The protein concentration was measured via a BCA protein assay kit (Thermo Scientific). The anti-Flag magnetic beads were washed three times with lysis buffer and co-incubated with the lysates at 4 °C overnight following the manufacturer’s protocol (Sigma M8823). The beads were subsequently washed three times with lysis buffer, incubated in 1× sample buffer (lysis buffer and 1× loading buffer). The protein lysates were heated for 5 min at 100 °C and separated via SDS‒PAGE. For the silver staining of proteins in polyacrylamide gels, the Fast Silver Stain kit was used according to the manufacturer’s protocol (Beyotime). The proteins corresponding to the bands on the gel were then identified through MS analysis.

### Immunoprecipitation

HEK293T cells were seeded in 10-cm dishes for 24 h before transfection. Co-transfection with 4 µg of Flag–NCOA7 and 4 µg of HA–G3BP1 (or LARP1, CAPRIN1, ATP6V1A, ATP6V1B2 and ATP6V1D), 4 µg of G3BP1 (or G3BP1^NTF2−^ and ATP6V1A) or 4 µg of NCOA7 (full-length or truncated versions) was performed via polyplus transfection following the manufacturer’s protocol. After 48 h, the cells were lysed and immunoprecipitation was performed. Flag beads were used for the immunoprecipitation and the samples were heated for 5 min at 95 °C and separated via SDS‒PAGE.

### SG induction and recovery

For SG formation, KGN cells or GCs isolated from mice or humans were treated with 300 µM arsenate for 1 h. For the SG recovery assay, the inducer (arsenate)-containing medium was removed, and the cells were cultured with fresh medium for 1 h or 4 h for recovery. ‘Stressed cell’ refers to a cell with one or more SGs and ‘SGs per cell’ refers to the number of SGs within the ‘stressed cells’^21,58^.

### SG separation

SGs were isolated from arsenate-treated KGN cells as described below. Following 1 h of treatment with 300 μM arsenate, cells were collected and centrifuged at 1,500*g* for 3 min. Cell pellets were resuspended in 1 ml of SG lysis buffer, lysed on ice using a 25G 5/8 needle and centrifuged at 1,000*g* for 5 min at 4 °C. The supernatant was further centrifuged at 18,000*g* for 20 min at 4 °C, after which the resulting pellets were resuspended in 1 ml of lysis buffer and subjected to another centrifugation at 18,000*g* for 20 min at 4 °C. Finally, the pellets were collected and resuspended in 300 μl of lysis buffer, followed by centrifugation at 850*g* for 2 min at 4 °C, yielding the enriched core fraction of SGs.

### Protein purification

Human NCOA7, G3BP1 and LC3B recombinant proteins were prepared as described below. First, the indicated genes were amplified via PCR, cloned and inserted into the pET.28a vector to produce His6-tag-fused recombinant proteins. For NCOA7 and G3BP1 was additionally fused to the 3′ end of the target gene. The expression of all the recombinant proteins was induced in *E.* *coli* BL21-CodonPlus (DE3) by adding 0.5 mM IPTG for 18 h at 16 °C, and the proteins were subsequently collected by sedimentation. To purify His–NCOA7, His–G3BP1 and His–LC3B recombinant proteins, the *E.* *coli* cells were resuspended in binding buffer (50 mM Tris-HCl, pH 7.9, 500 mM NaCl and 10 mM imidazole), lysed with a high-pressure homogenizer and sedimented at 5,000*g* for 30 min to pellet the debris. The supernatant lysates were purified on Ni-NTA agarose beads (QIAGEN). After extensive washing with binding buffer, the proteins were eluted with elution buffer (50 mM Tris-HCl, pH 7.9, 500 mM NaCl and 250 mM imidazole), further purified with a HiPrep 26/60 Sephacryl S-200 HR column (GE Healthcare, 17-1195-01) on an AKTA purifier (GE Healthcare) and eluted with a buffer containing 25 mM HEPES, pH 7.5, 1 M NaCl and 1 mM dithiothreitol (DTT). All purified proteins were concentrated via centrifugation (Millipore) and then stored in aliquots at −80 °C.

### LLPS

The purified recombinant proteins were dissolved in buffer containing 25 mM HEPES (pH 7.5), 1 M NaCl and 1 mM DTT and then mixed at the desired ratio and concentration, as indicated in the figure. Next, the concentration of NaCl was adjusted to 500 mM with low-salt buffer (25 mM HEPES, pH 7.5 and 1 mM DTT). The mixture was subsequently centrifuged, after which the aggregated proteins were removed, and the NaCl concentration was adjusted to 150 mM with low-salt buffer to induce phase separation. Finally, bright-field images of the LLPS droplets were obtained on a glass slide. For the sedimentation assay, the samples were centrifuged at 10,000*g* for 5 min, after which the supernatant and pellet were separated into two parts. The pellet fraction was washed and thoroughly resuspended in buffer containing 25 mM HEPES (pH 7.5), 150 mM NaCl and 1 mM DTT.

### His pulldown

For His pulldown, His-LC3B recombinant protein was purified as described above and then immobilized in a Pierce Spin Column with a Pulldown PolyHis Protein: Protein Interaction kit (Pierce) following the manufacturer’s protocol. The columns were then mixed with lysate from the NCOA7–Flag-overexpressing HEK293T cell line. The mixture was incubated for 1 h at 4 °C and washed four times with 500 µl of wash buffer. Proteins were eluted with SDS sample buffer and analyzed by 15% SDS‒PAGE and immunoblotting with an anti-Flag antibody. Coomassie blue staining was used to visualize the LC3B band.

### Autophagosome isolation

KGN cells were transfected with the NCOA7–Flag plasmid for 48 h and subsequently exposed to 300 μM arsenate for 1 h. Following this treatment, cells were collected and lysed in HB1 lysis buffer. The lysates were subjected to sequential differential centrifugation at 3,000*g* and 25,000*g* to collect the total sediment. The 25,000*g* membrane pellet was resuspended in 0.25 ml of 1.25 M sucrose buffer and overlaid with 0.25 ml of 1.1 M and 0.2 ml of 0.25 M sucrose buffer. Centrifugation was performed at 120,000*g* for 2 h to separate two fractions, one at the interface between 0.25 M and 1.1 M sucrose (L fraction) and the other at the bottom (P fraction). The L fraction was collected and resuspended in 0.2 ml of 19% OptiPrep for a step gradient in which each solution contained 0.1 ml of 22.5%, 0.2 ml of 19% (sample), 0.18 ml of 16%, 0.18 ml of 12%, 0.2 ml of 8%, 0.1 ml of 5% and 0.04 ml of 0% OptiPrep each. The OptiPrep gradient was centrifuged at 150,000*g* for 3 h and eight fractions, 0.1 ml each, were subsequently collected from the top. SDS loading buffer (1×) was added to the fractions, and immunoblotting was performed with the indicated antibodies.

### Autophagy activation and inhibition assay

In experiments to assess the autophagic clearance of SGs, cells were treated with 500 nM rapamycin for 48 h to induce autophagy or 50 µM chloroquine for 2 h to inhibit lysosomal activity before arsenate treatment. For the cellular senescence rescue experiments and cell counting assays, the cells were treated with 500 nM rapamycin on days 0, 2, 4 and 6, and SA-β-gal staining and cell counting were performed on days 4 and 8. Additionally, senescence marker genes and SASP genes were analyzed via RT‒qPCR on day 4.

### *NCOA7*-mRNA lipid nanoparticle construction and treatment

circ-*NCOA7* was purchased from Geneseed Biotech Corporation. The ionizable lipids PPZ-A10, DOPE, cholesterol and DMG-PEG 2000 were dissolved in ethanol at a molar ratio of 40:16:41.5:2.5, and the concentration of PPZ-A10 was 5 mg ml^−1^. The mRNA was diluted in citrate disodium hydrogen phosphate buffer (pH 4). LNPs were constructed by rapidly mixing the ethanol phase and aqueous phase via a microfluidic device and were subsequently dialyzed in DEPC-PBS solution (MWCO 100 kDa) for 12 h to remove ethanol and free mRNA. Before dialysis, the Quant-iT RiboGreen RNA kit mixture was mixed with LNPs or LNPs demulsified with 2% Triton X-100. With excitation by a laser at 480 nm, the fluorescence intensity at 520 nm was detected by a multimode plate reader (EnSight, PerkinElmer) and the mRNA content was calculated according to a standard curve.

For in vitro *N7* mRNA-LNP treatment, human GCs were cultured in six-well plates. Next, cells were incubated with fresh medium containing 150 ng ml^−1^ mRNA-LNPs on days 0 and 2. Subsequently, SA-β-gal staining and cell count assays were performed on days 4 and 8.

### Statistics and reproducibility

Data collection and analysis were not performed blind to the conditions of the experiments. Data collection and animal group assignment were both randomized. Data analyses were conducted using GraphPad Prism v.10.0 and ImageJ v.1.8.0 was used to quantify the image data. The Shapiro–Wilk test and quantile–quantile plot were combined to test for normality. Continuous variables with a normal distribution are presented as mean ± s.e.m. and compared using a two-tailed Student’s *t*-test or one-way analysis of variance. Non-normally distributed continuous variables were compared using a Mann–Whitney *U*-test and presented as the median (interquartile range). Sample sizes were chosen based on previous experiments and no statistical method was used to predetermine sample size for all experiments. No data were excluded from the analyses. Unless otherwise indicated, all the experiments were independently repeated at least three times with consistent conclusions, and representative images or data are shown. For each experiment, the appropriate controls used were as follows: control for POI and aged; DMSO for arsenate; wild-type for mutants or domain-truncated; wild-type for KO or *Ncoa7*^−*/*−^; flox/flox for *Ncoa7*-cKO; KO for rescue (#); si-*NC* cells for si-*NCOA7*, si-*ATG7*, si-*ATG9A* and si-*ATG13*; DMSO or scrambled mRNA-LNP for treatment with chloroquine, Rapa or *N7* mRNA-LNP. *P* < 0.05 was considered to indicate statistical significance (* or #) and *P* < 0.01, *P* < 0.001 and *P* < 0.0001 were considered highly statistically significant (**, *** and ****, or ##, ### and ####).

### Reporting summary

Further information on research design is available in the Nature Portfolio Reporting Summary linked to this article.

## Supplementary information

Reporting Summary

Supplementary Table Supplementary Table 1. Variants of NCOA7 gene identified in POI patients and their clinical characteristics. Supplementary Table 2. Clinical characteristics of control, POI and middle-aged participants. Supplementary Table 3.Interaction patterns between V-ATPase and NCOA7. Supplementary Table 4. Primers, siRNAs and sgRNAs. Supplementary Table 5. Antibodies. Supplementary Table 6. DEGs from the RNA-seq.

## Source data

Source Data Figs. 1–7 Statistical Source Data.

Source Data Extended Data Figs. 1–8 Statistical Source Data.

Source Data Fig. 1 Unprocessed western blots.

Source Data Fig. 2 Unprocessed western blots.

Source Data Fig. 3 Unprocessed western blots.

Source Data Fig. 4 Unprocessed western blots.

Source Data Fig. 5 Unprocessed western blots and gels.

Source Data Extended Data Fig.1 Unprocessed gels.

Source Data Extended Data Fig.2 Unprocessed western blots.

Source Data Extended Data Fig.3 Unprocessed western blots and gels.

Source Data Extended Data Fig.4 Unprocessed western blots and gels.

Source Data Extended Data Fig.5 Unprocessed western blots.

Source Data Extended Data Fig.6 Unprocessed western blots.

Source Data Extended Data Fig.7 Unprocessed western blots.

Source Data Extended Data Fig.8 Unprocessed western blots.

## Data Availability

CUT&Tag and RNA-seq data are available in the Gene Expression Omnibus (GEO) under accession numbers GSE250222 and GSE296744. Other data underlying this study are available as Source Data files and from the corresponding author upon reasonable request. Source Data are provided with the paper.
