# Supplementary material for: Stress granule clearance mediated by V-ATPase-interacting protein NCOA7 mitigates ovarian aging
Source: Nat Aging. 2025 Jul 31;5(8):1548–67. doi: 10.1038/s43587-025-00927-w (PMC12350179; doi:10.1038/s43587-025-00927-w)
Supplement: Supplementary file 1 — Reporting Summary [file 43587_2025_927_MOESM1_ESM.pdf]

nature portfolio

Corresponding author(s): Xue Jiao

Last updated by author(s): May 7, 2025

Reporting Summary

Nature Portfolio wishes to improve the reproducibility of the work that we publish. This form provides structure for consistency and transparency in reporting. For further information on Nature Portfolio policies, see our Editorial Policies and the Editorial Policy Checklist.

Statistics

For all statistical analyses, confirm that the following items are present in the figure legend, table legend, main text, or Methods section.

n/a	Confirmed	
☐	☒	The exact sample size (*n*) for each experimental group/condition, given as a discrete number and unit of measurement
☐	☒	A statement on whether measurements were taken from distinct samples or whether the same sample was measured repeatedly
☐	☒	The statistical test(s) used AND whether they are one- or two-sided<br>*Only common tests should be described solely by name; describe more complex techniques in the Methods section.*
☒	☐	A description of all covariates tested
☐	☒	A description of any assumptions or corrections, such as tests of normality and adjustment for multiple comparisons
☐	☒	A full description of the statistical parameters including central tendency (e.g. means) or other basic estimates (e.g. regression coefficient) AND variation (e.g. standard deviation) or associated estimates of uncertainty (e.g. confidence intervals)
☐	☒	For null hypothesis testing, the test statistic (e.g. *F*, *t*, *r*) with confidence intervals, effect sizes, degrees of freedom and *P* value noted<br>*Give P values as exact values whenever suitable.*
☒	☐	For Bayesian analysis, information on the choice of priors and Markov chain Monte Carlo settings
☒	☐	For hierarchical and complex designs, identification of the appropriate level for tests and full reporting of outcomes
☐	☒	Estimates of effect sizes (e.g. Cohen's *d*, Pearson's *r*), indicating how they were calculated

Our web collection on statistics for biologists contains articles on many of the points above.

Software and code

Policy information about availability of computer code

Data collection	Confocal microscopy image acquisition was performed with Imaris Viewer software10.2.0.<br>The three-dimensional structures of NCOA7 were constructed by AlphaFold 2.3.<br>Cell apoptosis was assessed using flow cytometry, and data were acquired using a BD LSRFortessa flow cytometer.
Data analysis	GraphPad Prism 10.0 was used for the statistical analysis and ImageJ 1.8.0 was used for quantifying image data.<br>Variations were predicted to be potentially deleterious by SIFT, PolyPhen-2, MutationTaster, CADD, DANN, and MetaSVM softwares.<br>The data from the apoptosis assay were analyzed using FACSDiva software 8.0 and FlowJo software 10.5.3.<br>All CUT&Tag data were mapped to the mm10 genome (GCF_000001635.26) using BWA v0.7.12. All low-quality reads and PCR adapters were removed. The BamCoverage command from deepTools 3.0.2 was used to generate the track files. All peak calling was performed with MACS2 2.1.2 using the options “macs2-q 0.05–call-summits–nomodel–shift-100–extsize 200–keep-dup all”. The genomic distribution of CUT&Tag peaks was annotated with the R package ChIPseeker. Heatmaps and metaplots were generated for the protein-coding genes or specific peaks using deepTools 3.0.2.

For manuscripts utilizing custom algorithms or software that are central to the research but not yet described in published literature, software must be made available to editors and reviewers. We strongly encourage code deposition in a community repository (e.g. GitHub). See the Nature Portfolio guidelines for submitting code & software for further information.

Data

Policy information about availability of data

All manuscripts must include a data availability statement. This statement should provide the following information, where applicable:

- Accession codes, unique identifiers, or web links for publicly available datasets
- A description of any restrictions on data availability
- For clinical datasets or third party data, please ensure that the statement adheres to our policy

The CUT&Tag and RNA-seq data are available in Gene Expression Omnibus (GEO) database with the accession number GSE250222 and GSE158526.

Research involving human participants, their data, or biological material

Policy information about studies with human participants or human data. See also policy information about sex, gender (identity/presentation), and sexual orientation and race, ethnicity and racism.

Reporting on sex and gender	Our findings only apply to one sex (female) because we studied "ovaries". All participants were recruited from the Hospital for Reproductive Medicine Affiliated to Shandong University and provided written informed consent.
Reporting on race, ethnicity, or other socially relevant groupings	The participants included in our study were all Chinese women, because the participants were recruited from the Hospital for Reproductive Medicine Affiliated to Shandong University, and the vast majority of women attending our hospital were residents of nearby Shandong, China.
Population characteristics	All participants were women with chromosomes 46, XX, categorized into three cohorts:<br>preclinical premature ovarian insufficiency group (age 33.81 ± 4.29 years);<br>advanced-aged premenopausal group (age 43.00 ± 2.05 years);<br>infertility with normal ovarian function group (age 32.30 ± 3.55 years).
Recruitment	All participants were recruited from the Hospital for Reproductive Medicine Affiliated with Shandong University from July 2021 to July 2024. The inclusion criteria for POI included secondary amenorrhea for at least 4 months and a serum basal FSH concentration > 25 IU/L (on two occasions > 1 month apart) before the age of 40 years, in accordance with the ESHRE and Chinese guidelines. Preclinical POI was defined as regular or irregular menses, elevated basal serum FSH (10 IU/L < FSH ≤ 25 IU/L, on two occasions > 4 weeks apart) and an antral follicle count (AFC) < 5 before the age of 40 years, as previously reported. The advanced-aged premenopausal (aged) group included women older than 40 years before menopause. Patients with regular menstrual cycles and normal FSH levels (<10 IU/L) who were seeking infertility treatment due to tubal obstruction or male factors were recruited as controls. Women with chromosomal abnormalities, known gene mutations, a history of ovarian surgery, radio- or chemotherapy, a history of recurrent spontaneous abortion, endometriosis or autoimmune disease, or infection in the previous three months were excluded.<br>Participants were recruited from a single hospital, which may introduce admission rate bias due to potential overrepresentation of certain patient profiles. However, the relatively large sample size mitigates this concern by maintaining statistical power.
Ethics oversight	All procedures involving patients in this study were approved by the Ethics Committee of the Hospital for Reproductive Medicine Affiliated to Shandong University.

Note that full information on the approval of the study protocol must also be provided in the manuscript.

Field-specific reporting

Please select the one below that is the best fit for your research. If you are not sure, read the appropriate sections before making your selection.

☒ Life sciences ☐ Behavioural & social sciences ☐ Ecological, evolutionary & environmental sciences

For a reference copy of the document with all sections, see nature.com/documents/nr-reporting-summary-flat.pdf

Life sciences study design

All studies must disclose on these points even when the disclosure is negative.

Sample size	No statistical methods were used to pre-determine sample sizes, but our sample size are similar to those reported previously (e.g. McHugh, et al. Nat Cell Biol. 2023; Guerrero, et al. Nat aging. 2022)
Data exclusions	No data were excluded from the analyses.
Replication	All the experiments were independently repeated at least three times with consistent conclusions.
Randomization	The allocation was random.

Blinding

Data collection and analysis were conducted without blinding to the experimental conditions. Multiple biological replicates and independent experiments to ensure reproducibility.

Reporting for specific materials, systems and methods

We require information from authors about some types of materials, experimental systems and methods used in many studies. Here, indicate whether each material, system or method listed is relevant to your study. If you are not sure if a list item applies to your research, read the appropriate section before selecting a response.

Materials & experimental systems

n/a	Involved in the study
☐	☒ Antibodies
☐	☒ Eukaryotic cell lines
☒	☐ Palaeontology and archaeology
☐	☒ Animals and other organisms
☒	☐ Clinical data
☒	☐ Dual use research of concern
☒	☐ Plants

Methods

n/a	Involved in the study
☒	☐ ChIP-seq
☒	☐ Flow cytometry
☒	☐ MRI-based neuroimaging

Antibodies

Antibodies used

Full list of antibodies can be found in Supplementary_Table.
Rabbit anti-NCOA7 Abcam Cat# ab254732 WB 1:3000; IF 1:100; IHC 1:300
Mouse anti-GM130 Abcam Cat# ab169276 WB 1:2000; IF 1:300
Mouse anti-G3BP1 Proteintech Cat# 66486-1 WB 1:8000; IF 1:300
G3BP1 Antibody (1E4A2), CoraLite® 594 Proteintech Cat# CL594-66486 IF 1:500
Rabbit anti-ATP6V1A Abcam Cat# ab137574 WB 1:5000; IF 1:500
ATP6V1B2 antibody Proteintech Cat# 68441-1-PBS WB 1:8000
Rabbit anti-CAPRIN1 Proteintech Cat# 15112-1-AP IF 1:500
Rabbit anti-CDKN2A/p16INK4a Abclonal Cat# A0262 WB 1:1000; IHC 1:100
Rabbit anti-CDKN1A/p21Cip1 Abclonal Cat# A19094 WB 1:1000; IHC 1:100
Rabbit anti-LAMP1 Abcam Cat# ab25245 WB 1:1000
Rabbit anti-ATG7 Proteintech Cat# 10088-2-AP WB 1:1500
Rabbit anti-ATG9A Abcam Cat# ab108338 WB 1:1000
Rabbit anti-ATG13 Proteintech Cat# 18258-1-AP WB 1:800
Rabbit anti-FLAG CST Cat# 14793 WB 1:10000
Rabbit anti-HA CST Cat# 3724 WB 1:10000
Mouse anti-Myc CST Cat# 2276 WB 1:10000
Mouse anti-GAPDH Abmart Cat# M20006 WB 1:8000
Rabbit anti-β-Actin Affinity Cat# AF7018 WB 1:10000
Rabbit anti-α-Tubulin Affinity Cat# AF7010 WB 1:8000
Mouse anti-p62 Abcam Cat# ab56416 WB 1:3000
Rabbit anti-LC3B Sigma Cat# L7543 WB 1:3000
Rat anti-LAMP1 Abcam Cat# ab25245 IF 1:500
Goat anti-Rabbit IgG (H+L) Cross-Adsorbed Secondary Antibody, Alexa Fluor™ 488 Invitrogen Cat# A-11008 IF 1:10000
Rhodamine(TRITC)AffiniPure Goat Anti-Rabbit IgG(H+L) Yeasen Cat# 33109ES60 IF 1:300
FITC-AffiniPure Goat Anti-Mouse IgG(H+L) Yeasen Cat# 33207ES60 IF 1:300
Donkey anti-Mouse IgG (H+L) Highly Cross-Adsorbed Secondary Antibody, Alexa Fluor™ 568 Invitrogen Cat# A10037 IF 1:10000
Goat anti-Rat IgG H&L- Adsorbed Secondary Antibody Alexa Fluor® 647 Abcam Cat# ab150167 IF 1:500
Donkey anti-Mouse IgG (H+L) Highly Cross-Adsorbed Secondary Antibody, Alexa Fluor™ 647 Invitrogen Cat# A-31571 IF 1:500
Goat Anti-Rabbit IgG H&L (HRP) Abcam Cat# ab6721 WB 1:10000
Goat Anti-Mouse IgG H&L (HRP) Abcam Cat# ab6789 WB 1:10000

Validation

Rabbit anti-NCOA7; Suitable for: IF, IHC, WB; Reacts with: human, mouse
Mouse anti-GM130; Suitable for: WB, IF; Reacts with: human, mouse
Mouse anti-G3BP1; Suitable for: WB, IF, IHC; Reacts with: human, mouse, rat
Rabbit anti-ATP6V1A; Suitable for: WB, IF; Reacts with: human, mouse
Rabbit anti-CAPRIN1; Suitable for: WB, IF; Reacts with: human, mouse
Rabbit anti-CDKN2A/p16INK4a; Suitable for: WB, IF; Reacts with: human, mouse
Rabbit anti-CDKN1A/p21Cip1; Suitable for: WB, IF; Reacts with: human, mouse
Rabbit anti-LAMP1; Suitable for: WB, IF; Reacts with: human, mouse
Rabbit anti-ATG7; Suitable for: WB, IF; Reacts with: human, mouse
Rabbit anti-ATG9A; Suitable for: WB, IF; Reacts with: human, mouse
Rabbit anti-ATG13; Suitable for: WB, IF; Reacts with: human, mouse
Rabbit anti-FLAG; Suitable for: WB, IF, IP, CHIP; Reacts with: human, mouse, rat
Rabbit anti-HA; Suitable for: WB, IF, IP, CHIP; Reacts with: human, mouse, rat
Mouse anti-Myc; Suitable for: WB, IF, IP, CHIP; Reacts with: human, mouse, rat

Mouse anti-GAPDH; Suitable for: WB, IF, IP, CHIP; Reacts with: human, mouse, rat
Rabbit anti-β-Actin; Suitable for: WB, IF, IP, CHIP; Reacts with: human, mouse, rat
Rabbit anti-α-Tubulin; Suitable for: WB, IF, IP, CHIP; Reacts with: human, mouse, rat
Mouse anti-p62; Suitable for: IHC-P, IP, WB, ICC/IF, Flow Cyt; Reacts with: human
Rabbit anti-LC3B Sigma Cat# L7543; Suitable for: IHC-P, WB; Reacts with: human, mouse, rat

Eukaryotic cell lines

Policy information about cell lines and Sex and Gender in Research

Cell line source(s)	The KGN cell line (RCB1154) was obtained from the RIKEN BioResource Center (Japan); the HEK293T cell line (3101HUMSCSP502) was sourced from the Beijing Institute of Medical Research (BMCR, China); and the HeLa cell line was acquired from the Cell Resource Center, Institute of Basic Medical Sciences (IBMS), Chinese Academy of Medical Sciences/ Peking Union Medical College (CAMS/PUMC, China).
Authentication	KGN cells were authenticated by immunofluorescence using antibodies against CYP19A1 and FSHR, with >95% positivity confirming cellular purity. HEK293T and HeLa cell lines, sourced from an authoritative cell bank, were further validated via morphological analysis.
Mycoplasma contamination	All the cell cultures were tested negative for mycoplasma contamination.
Commonly misidentified lines (See ICLAC register)	The study did not involve misidentified cell lines.

Animals and other research organisms

Policy information about studies involving animals; ARRIVE guidelines recommended for reporting animal research, and Sex and Gender in Research

Laboratory animals	WT C57BL/6J mice were purchased from Beijing Vital River Laboratory, and C57BL/6J-Ncoa7-/- mice, developed using CRISPR/Cas9 technology, were obtained from Cyagen Bioscience. The Ncoa7flox/flox mice were purchased from Gempharmatech Co., Ltd. The Foxl2Cre knock-in mice were obtained from Dr. Fei Gao, the Institute of Zoology, Chinese Academy of Sciences (Beijing, China). The Foxl2Cre mice were crossed with Ncoa7flox/flox mice to generate Foxl2cre; Ncoa7flox/flox mice. Mice were bred and raised for observation from 8 weeks of age until 10 months of age. The ages of mice performing the experiments were illustrated in the figure legends.<br>All mice were housed on a 12-h light/dark cycle at 25 ℃ and were provided with standard chow and water ad libitum.
Wild animals	The study did not involve wild animals.
Reporting on sex	Our findings only apply to one sex (female) because we studied "ovaries".
Field-collected samples	The study did not involve samples collected from the field.
Ethics oversight	The animal protocols and experiments were approved by the Institutional Animal Care and Use Committees of Shandong University (SDU-IACUC-20220003) and were performed in accordance with the relevant guidelines.

Note that full information on the approval of the study protocol must also be provided in the manuscript.

Plants

Seed stocks	Plants were not used in this study.
Novel plant genotypes	Plants were not used in this study.
Authentication	Plants were not used in this study.
