# Supplementary figures and images for: Stress granule clearance mediated by V-ATPase-interacting protein NCOA7 mitigates ovarian aging
Source: Nat Aging. 2025 Jul 31;5(8):1548–67. doi: 10.1038/s43587-025-00927-w (PMC12350179; doi:10.1038/s43587-025-00927-w)

Figure1e

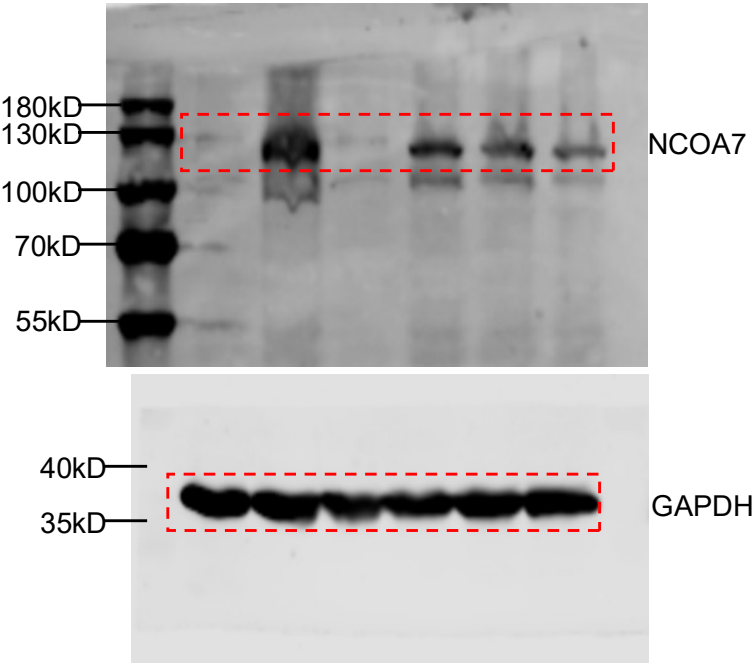

Figure1g

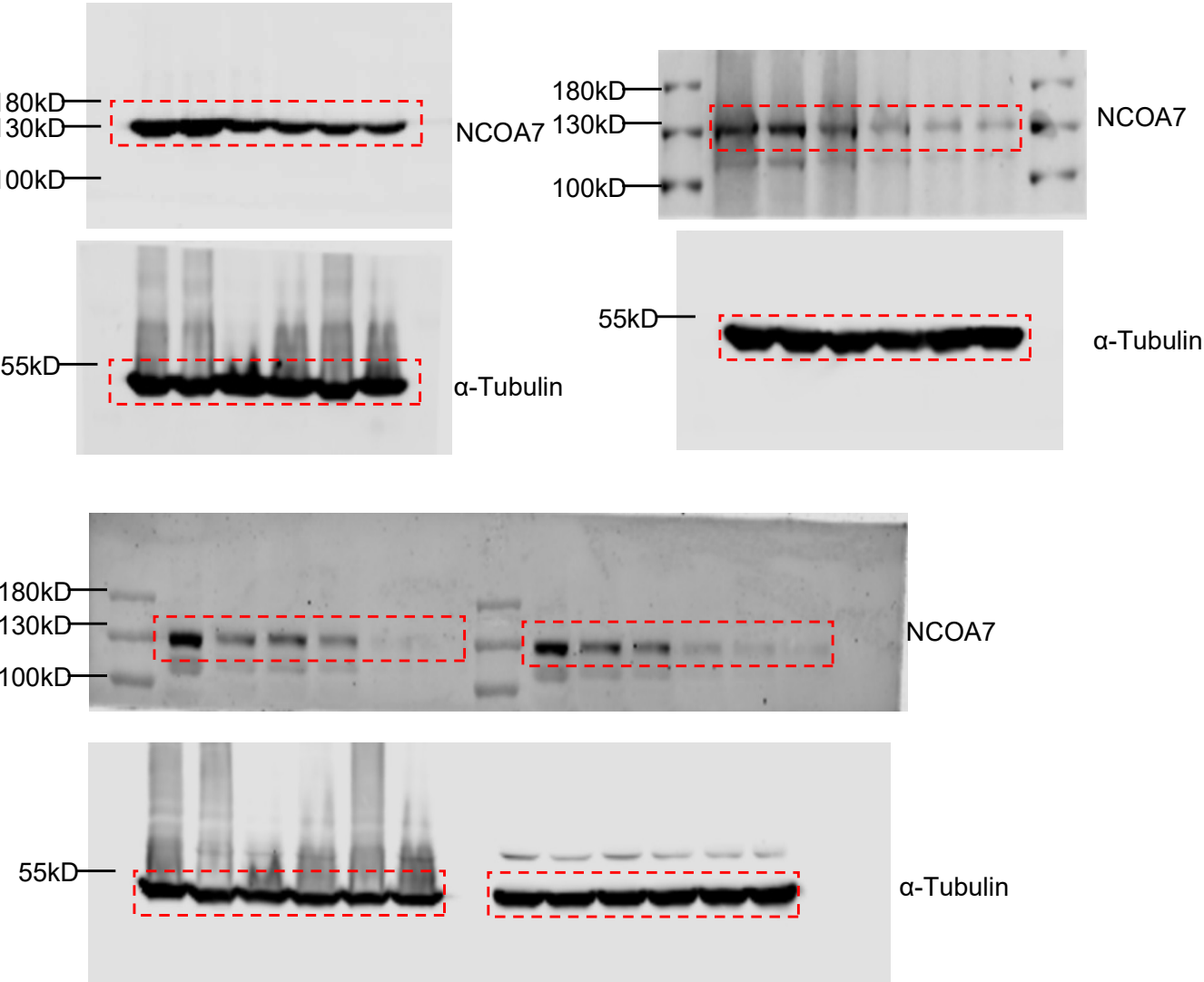

Supplement: Supplementary file 5 — Unprocessed western blots. [file 43587_2025_927_MOESM5_ESM.pdf]

**Figure2c**

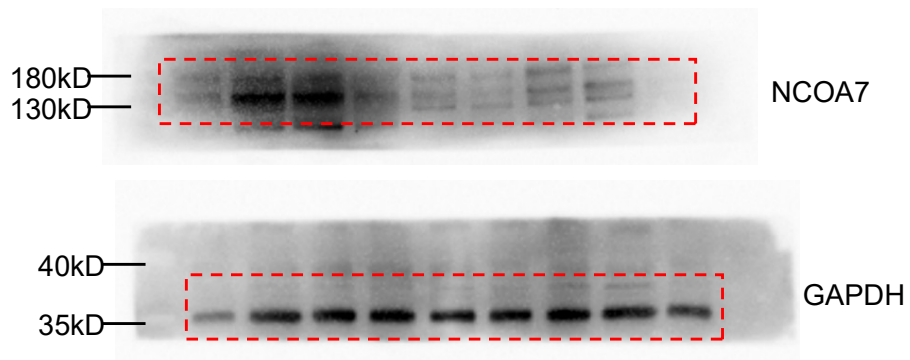

Supplement: Supplementary file 6 — Unprocessed western blots. [file 43587_2025_927_MOESM6_ESM.pdf]

**Figure3b**

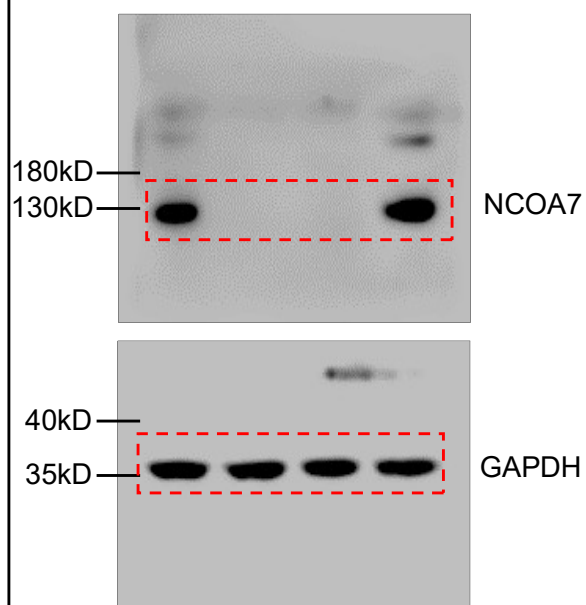

Supplement: Supplementary file 7 — Unprocessed western blots. [file 43587_2025_927_MOESM7_ESM.pdf]

**Figure4b**

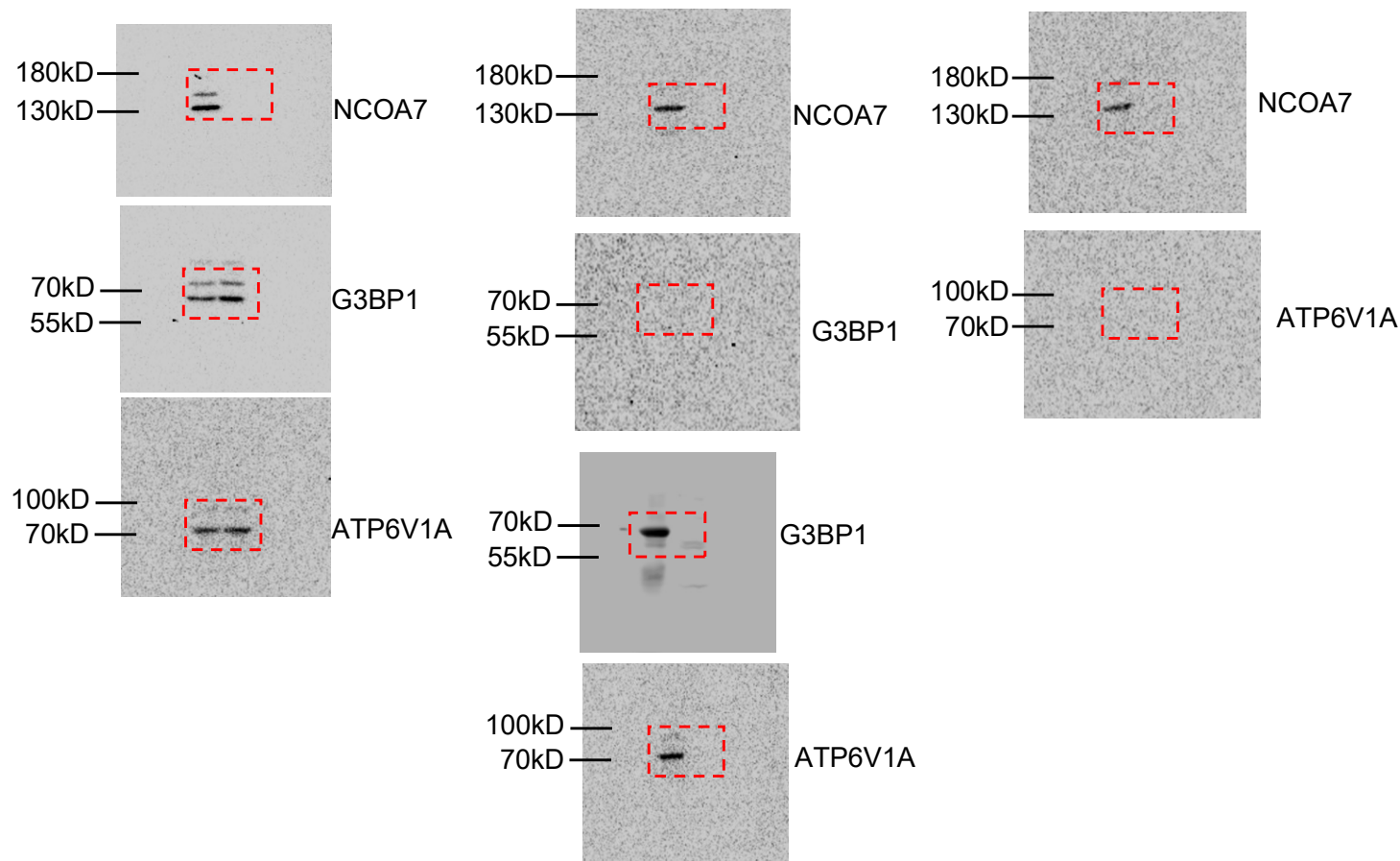

**Figure4g**

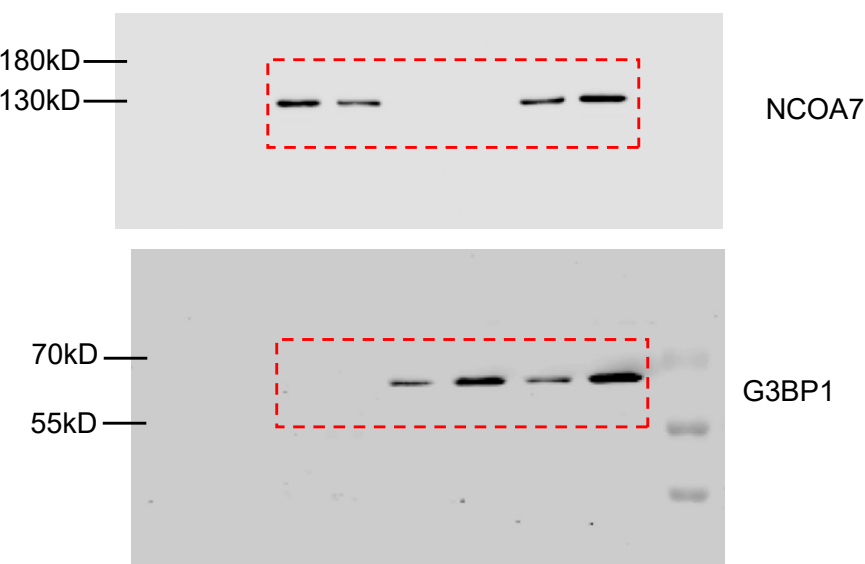

Figure4i

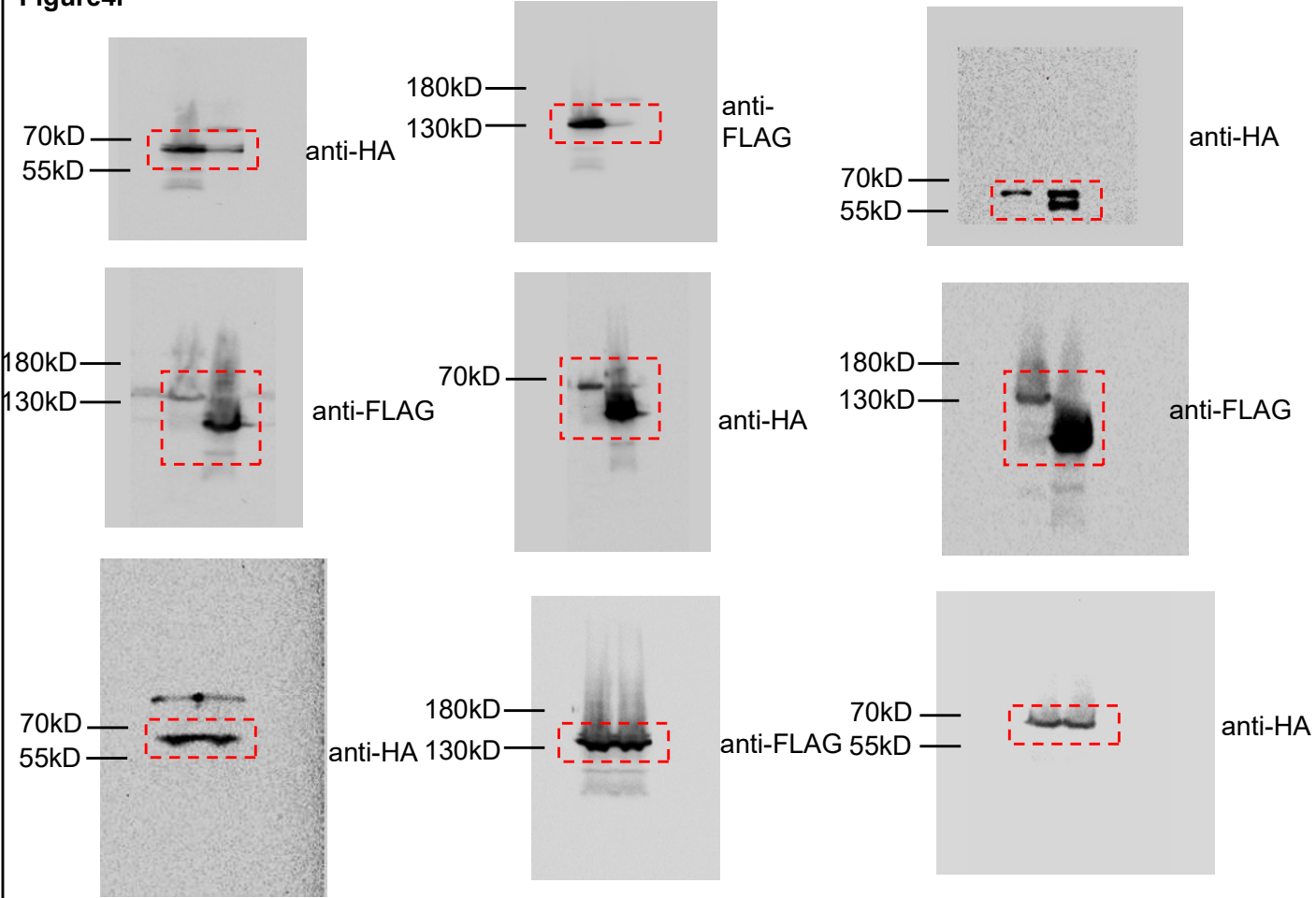

Supplement: Supplementary file 8 — Unprocessed western blots. [file 43587_2025_927_MOESM8_ESM.pdf]

**Figure5c**

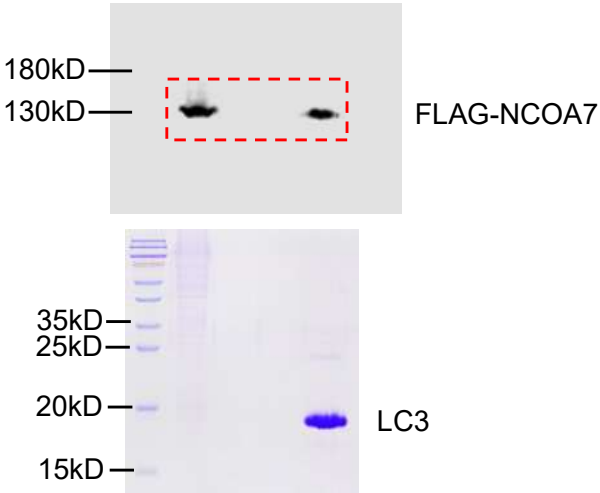

Supplement: Supplementary file 9 — Unprocessed western blots and gels. [file 43587_2025_927_MOESM9_ESM.pdf]

Extended Data Fig.1

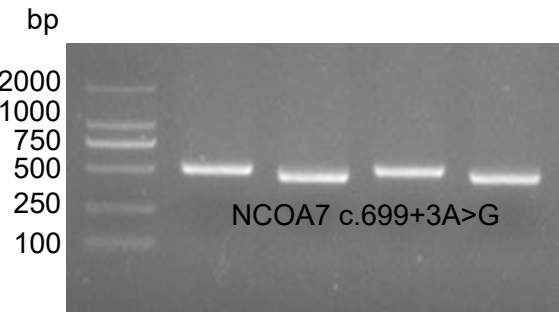

Supplement: Supplementary file 10 — Unprocessed gels. [file 43587_2025_927_MOESM10_ESM.pdf]

**Extended Data Fig.2b**

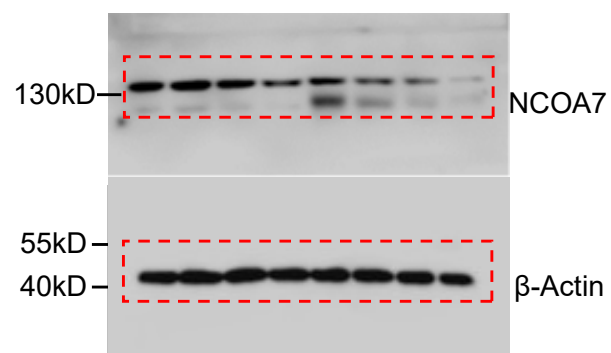

**Extended Data Fig.2d**

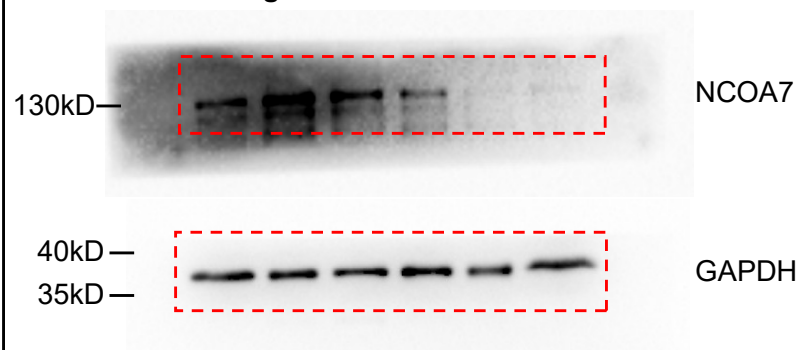

**Extended Data Fig.2h**

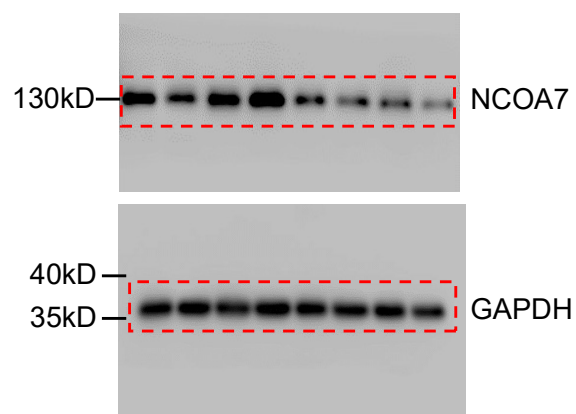

Supplement: Supplementary file 11 — Unprocessed western blots. [file 43587_2025_927_MOESM11_ESM.pdf]

**Extended Data Fig.3d**

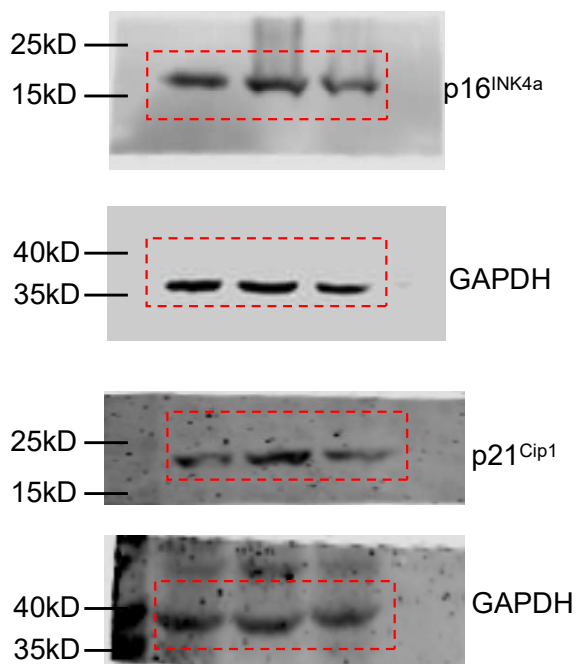

**Extended Data Fig.3e**

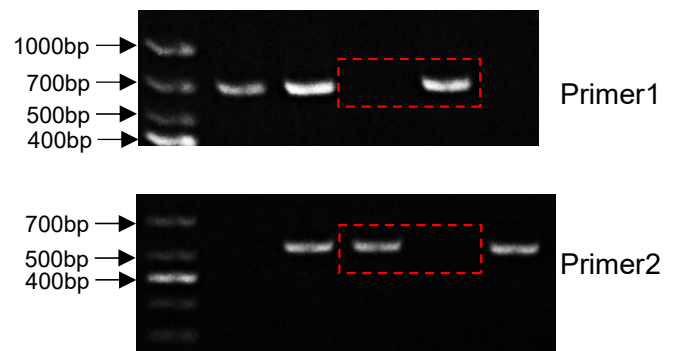

**Extended Data Fig.3f**

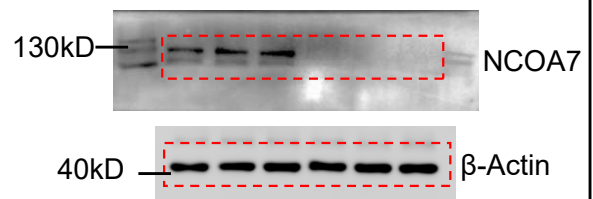

Supplement: Supplementary file 12 — Unprocessed western blots and gels. [file 43587_2025_927_MOESM12_ESM.pdf]

**Extended Data Fig.4a**

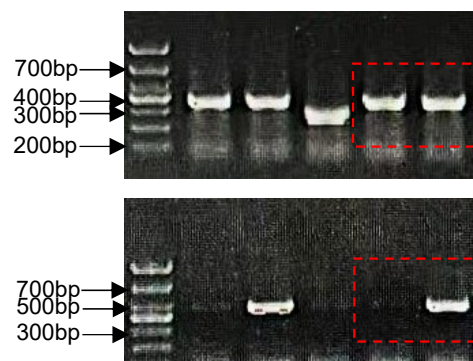

**Extended Data Fig.4c**

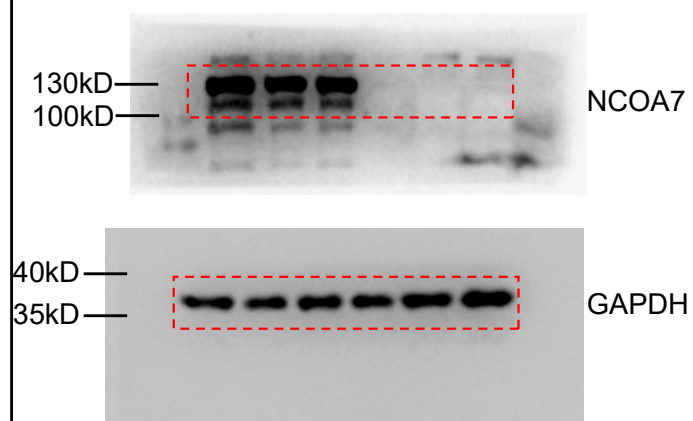

Supplement: Supplementary file 13 — Unprocessed western blots and gels. [file 43587_2025_927_MOESM13_ESM.pdf]

Extended Data Fig.6e

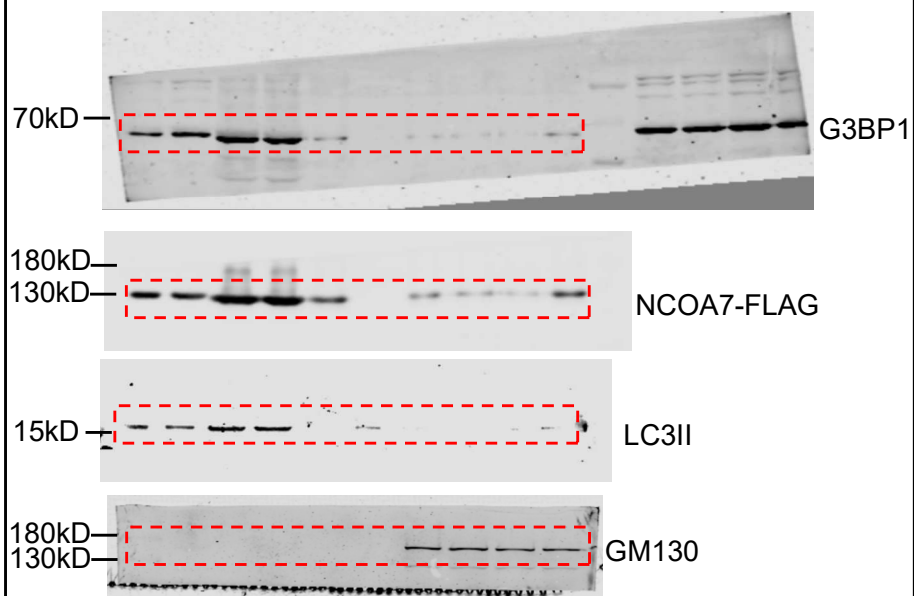

Extended Data Fig.6h

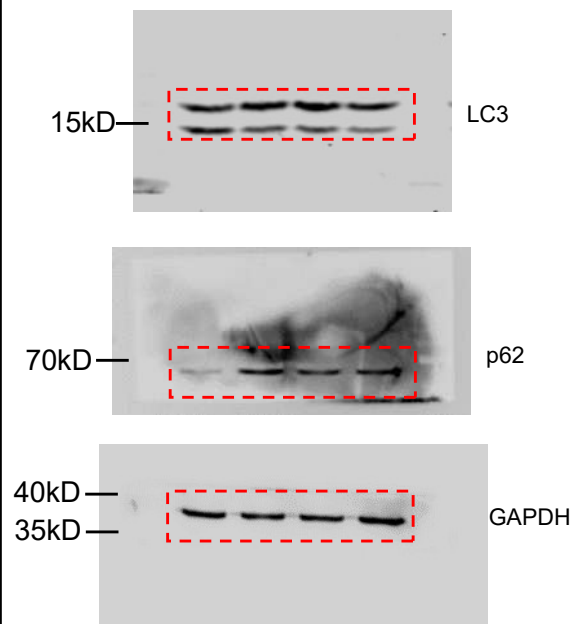

Extended Data Fig.6f

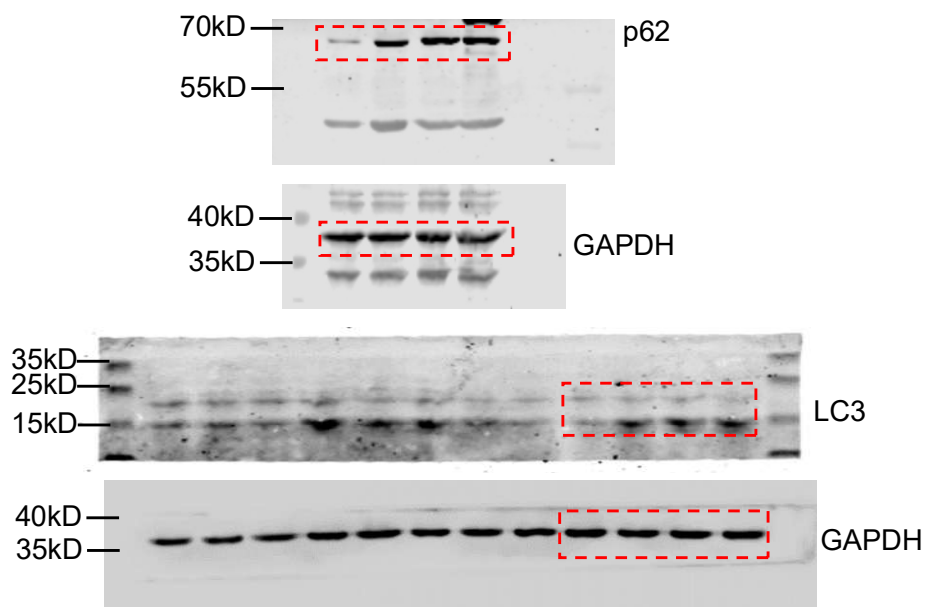

Extended Data Fig.6g

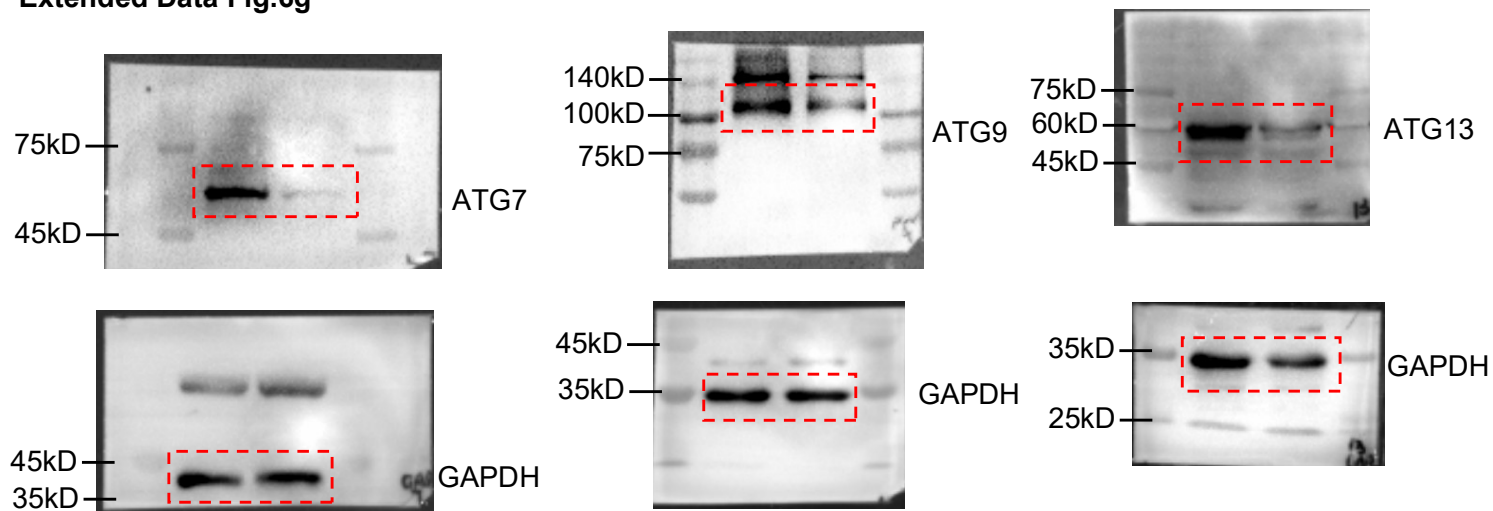

Supplement: Supplementary file 15 — Unprocessed western blots. [file 43587_2025_927_MOESM15_ESM.pdf]

### Extended Data Fig.7a

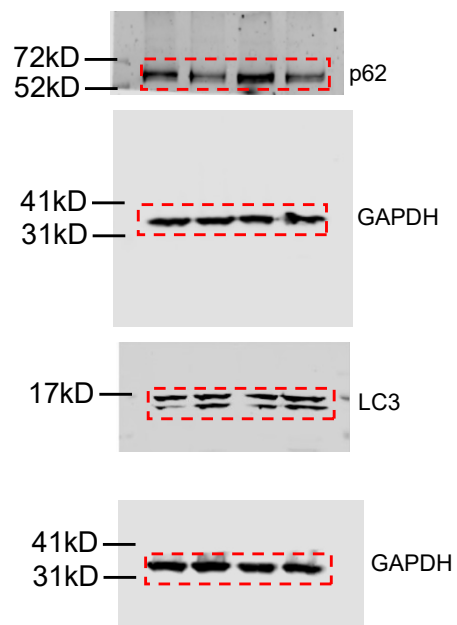

Supplement: Supplementary file 16 — Unprocessed western blots. [file 43587_2025_927_MOESM16_ESM.pdf]

**Extended Data Fig.8b**

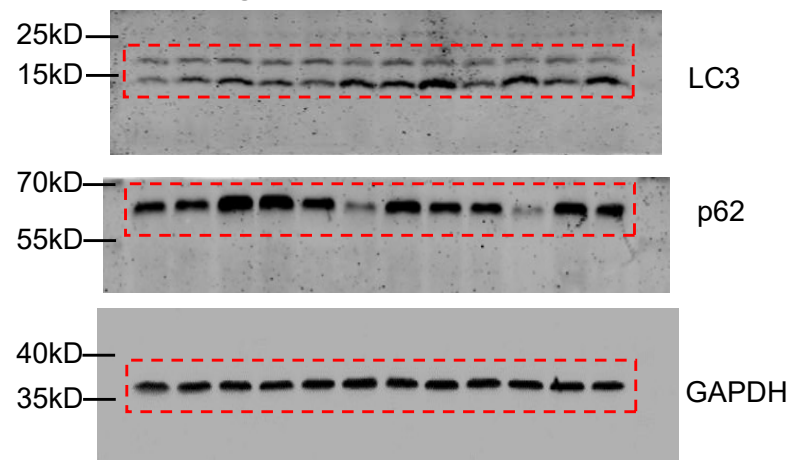

**Extended Data Fig.8f**

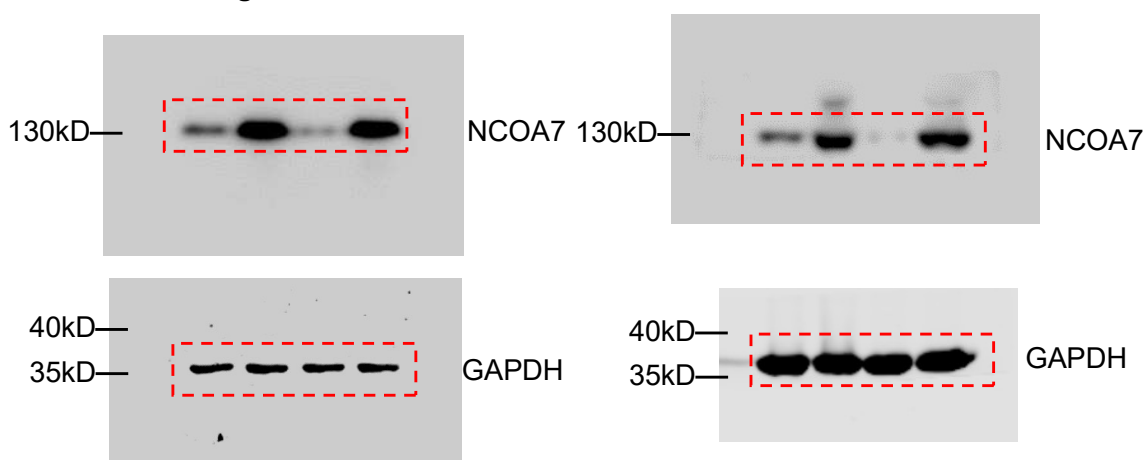

Supplement: Supplementary file 17 — Unprocessed western blots. [file 43587_2025_927_MOESM17_ESM.pdf]
