# Supplementary material for: Stress granule clearance mediated by V-ATPase-interacting protein NCOA7 mitigates ovarian aging
Source: Nat Aging. 2025 Jul 31;5(8):1548–67. doi: 10.1038/s43587-025-00927-w (PMC12350179; doi:10.1038/s43587-025-00927-w)

Extended Data Fig.5b

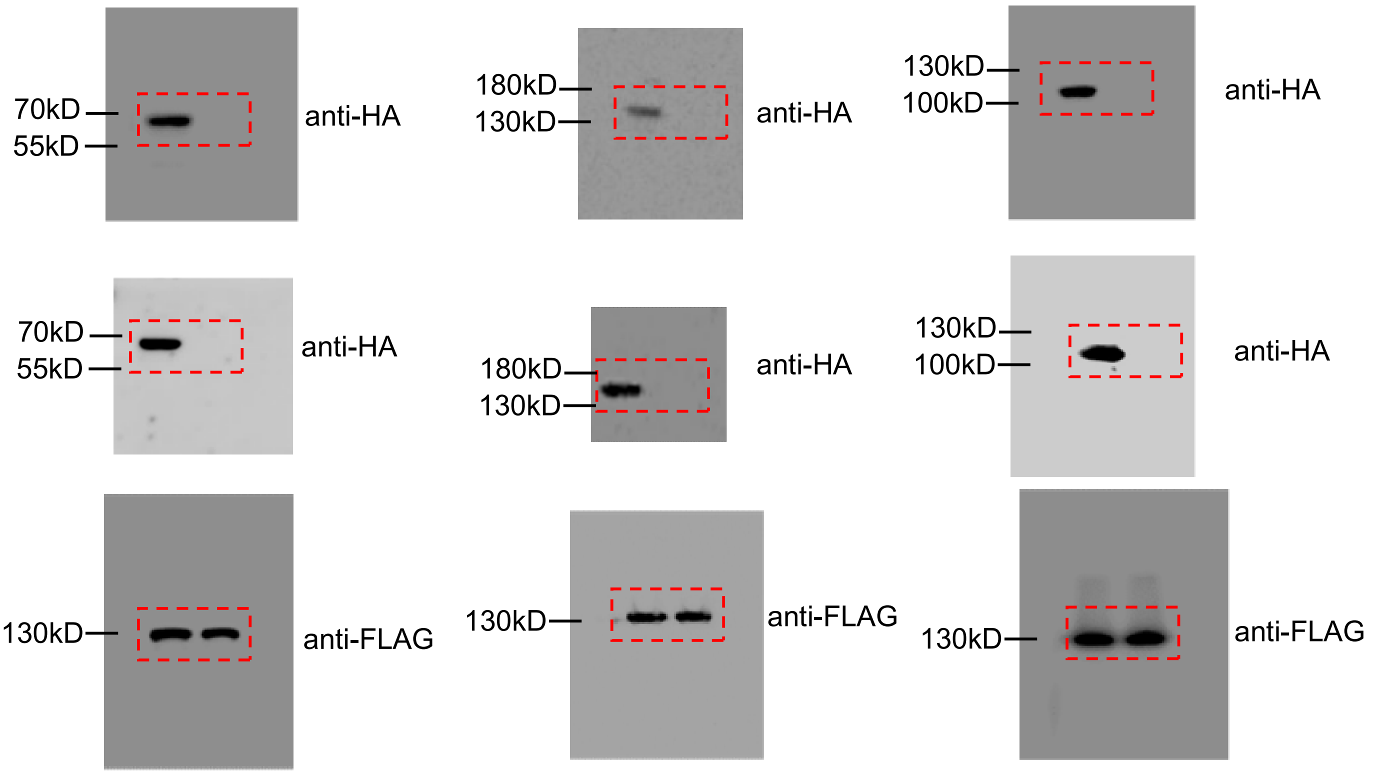

Extended Data Fig.5c

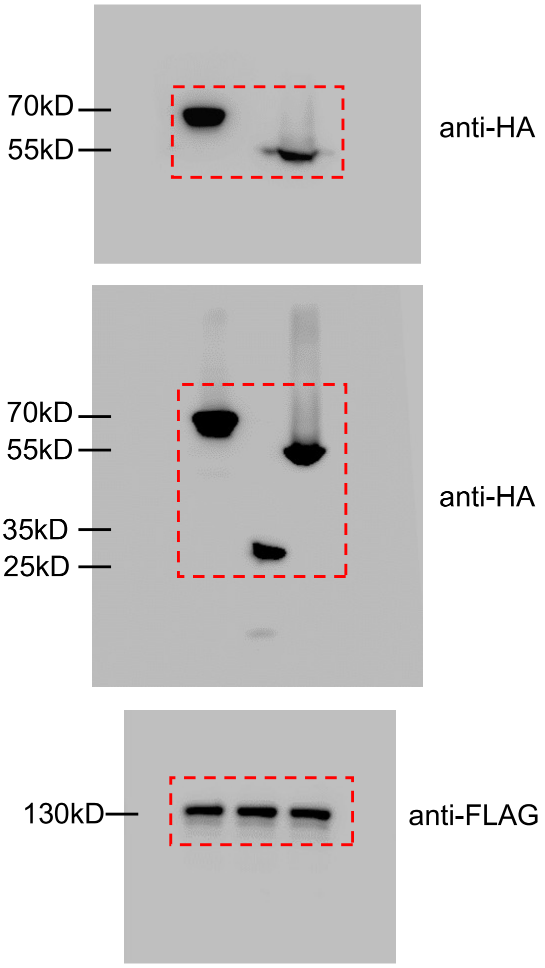

Extended Data Fig.5e

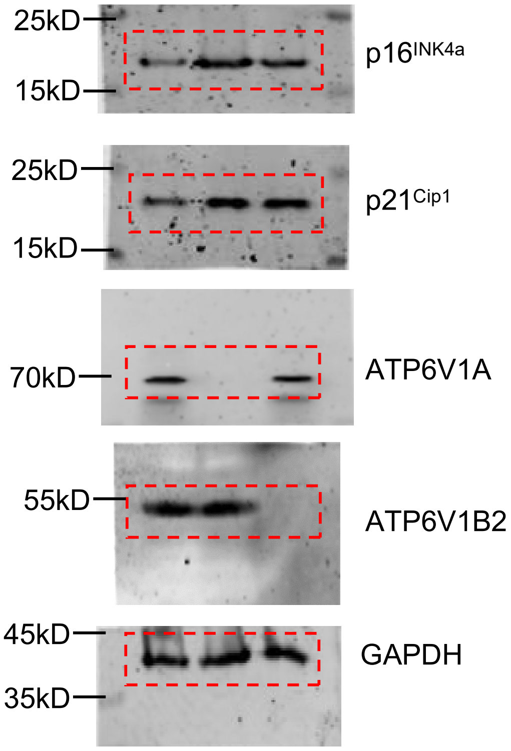

Extended Data Fig.5f

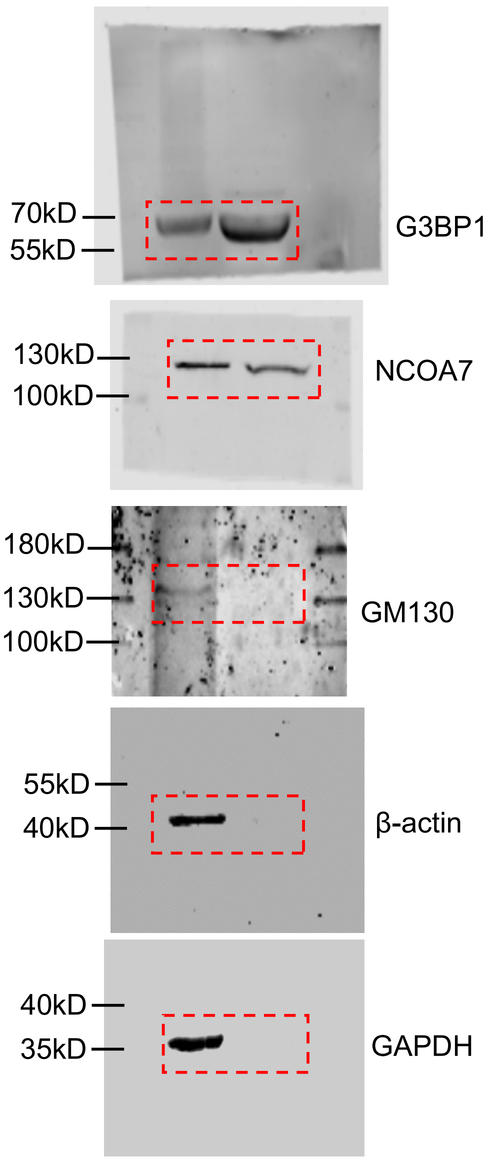

Extended Data Fig.5l

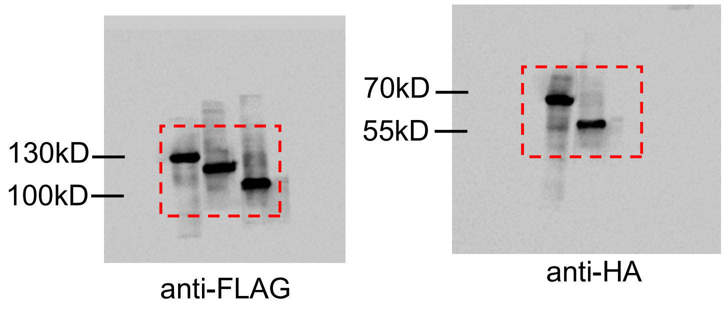

Extended Data Fig.5m

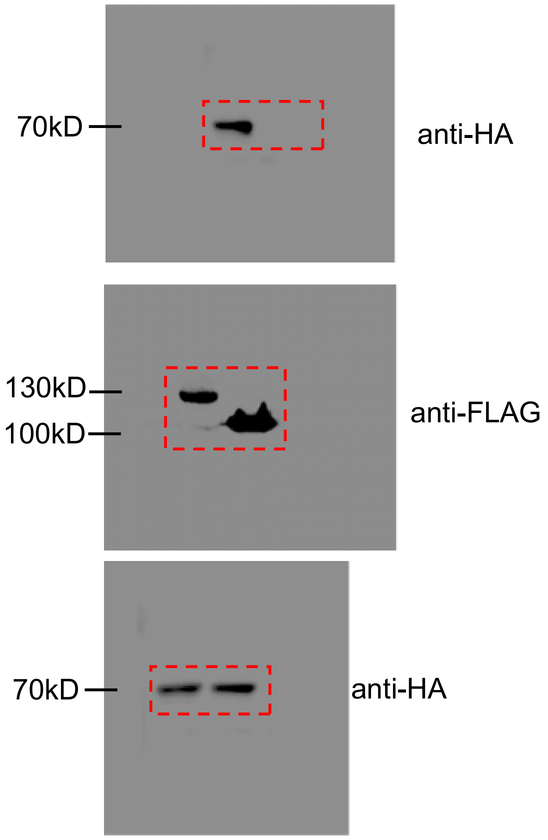

Supplement: Supplementary file 14 — Unprocessed western blots. [file 43587_2025_927_MOESM14_ESM.pdf]
